# Genomic insights into novel predatory myxobacteria isolated from human feces

**DOI:** 10.1128/spectrum.02147-24

**Published:** 2025-05-22

**Authors:** Joyasree Das, Shilpee Pal, Anu Negi, Shiva S. Sundharam, Amit Yadav, Srikrishna Subramanian, S. K. Sinha, Jayanta Samanta, Srinivasan Krishnamurthi

**Affiliations:** 1 Microbial Type Culture Collection & Gene Bank (MTCC), CSIR-Institute of Microbial Technology 29748, Chandigarh, India; 2 Bioinformatics Centre (BIC), CSIR-Institute of Microbial Technology 29748, Chandigarh, India; 3 Academy of Scientific and Innovative Research (AcSIR), CSIR-HRDC Campus 201510 , Ghaziabad, India; 4 Department of Gastroenterology, Postgraduate Institute of Medical Education and Research (PGIMER), Chandigarh, India; Lerner Research Institute, Cleveland, Ohio, USA

**Keywords:** myxobacteria, human gut, *Myxococcus*, genomics, metabarcoding, phylogenetic analysis, phylogenomics

## Abstract

Myxobacteria are Gram-negative, spore-forming predatory bacteria isolated from diverse environmental samples that feed on other microbes for their survival and growth. However, no reports of cultured representatives from the human gut have been published to date, although previous investigations have revealed the presence of myxobacterial operational taxonomic units (OTUs) in skin and fecal samples. In this study, three myxobacterial strains designated as O35, O15, and Y35 were isolated and purified from fecal samples of two inflammatory bowel disease (IBD) patients. The 16S rRNA gene sequence analysis and phylogeny identified the strains as *Myxococcus* spp. belonging to two different clades. Genome-based phylogeny and overall genome-related indices, i.e., average amino acid identity and percentage of conserved proteins, confirmed the heterogeneity within the genus and placed the three strains within two different clades separated at the level of different genera. Digital DNA-DNA hybridization and average nucleotide identity values indicated that they belonged to two novel *Myxococcus* spp. The analysis of meta-barcoding data from IBD and control cohorts detected OTU lineages closely affiliated to the three novel strains. Based on evidence from detailed structural and functional genomics, we propose the novel species *Myxococcus faecalis* sp. nov. O35^T^ and a new genus *Pseudomyxococcus* gen. nov. to accommodate the novel species *Pseudomyxococcus flavus* sp. nov. Y35^T^. Overall, these findings provide new information about the occurrence of myxobacteria in the human gut and lay the foundations for a new classification scheme for myxobacterial taxa.

**IMPORTANCE:**

Myxobacteria have been described from a variety of niches ranging from terrestrial to marine habitats and are known to harbor a diverse portfolio of bioactive molecules. However, to date, there has been no report of isolating culturable representatives from the human gut. This study describes novel myxobacteria from the human gut based on phylogenomics and phenotypic description. The findings are complemented by sequence-based data, wherein operational taxonomic unit (OTU) lineages closely affiliated with the isolated strains have been identified, thus opening a Pandora's box of opportunities for research into the microbial ecology and functional potential of these taxa in the gut ecosystem. Additionally, the study also seeks to establish a new systematic framework, expanding our understanding of myxobacterial taxonomy.

## INTRODUCTION

Myxobacteria are facultative predatory microorganisms that prey upon various Gram-positive and Gram-negative bacteria with their “wolf-pack hunting” strategy (1). They generally contain genomes of big size (4 to 16 Mb) and display fascinating physiological features such as swarming motility and fruiting body formation. They have an inherent ability to synthesize a variety of secondary metabolites that has attracted researchers toward their intensive study (2–9).

Application of novel culturing methodologies and culture-independent approaches, including clone-based and metagenomics analyses, has revealed the presence of novel lineages in the marine myxobacteria cluster and *Sandaracinaceae*-related cluster. These novel lineages were identified from deep-sea sediment samples (10) and seawater, seafloor/mangrove sediments, marine cyanobacterial blooms, corals, haloalkaline soil, hypersaline microbial mats, and salt marsh sediments (10-13). Phylogenetic analysis shows these lineages were separated from well-known sub-orders *Nannocystineae*, *Sorangiineae*, and *Cystobacterineae* that majorly harbor cultured representatives (10–13). Some human microbiome studies have suggested the existence of myxobacterial operational taxonomic units (OTUs) in fecal samples from inflammatory disease patients and animal models (6, 14). Thus, it would be interesting to ascertain their role in gut homeostasis and dysbiosis (if any) associated with several disorders, i.e., inflammatory bowel disease (IBD) (15–17) and/or celiac disease (CD) (18, 19). Like in other habitats, myxobacteria might play an ecological equalizer role in controlling the human gut microbiome, as evidenced by the presence of predatory gut proteobacteria such as *Bdellovibrio* (*B. bacteriovorus*), which is a part of a healthy human gut and has a role in controlling gut pathogens (20–22). Like *Bdellovibrio*, myxobacteria have been isolated from the dung of various herbivores, including deer, horses, goats, and pigs; however, to date, axenic myxobacterial strains from the human gut have not been reported. This is likely due to difficulty in cultivation and ignorance regarding their functional role in this ecosystem.

Furthermore, there is an inadequate understanding of myxobacterial classification due to comparatively complex systematics involving fruiting body formation, slow growth rates, and the inability to achieve species/genus resolution through single-gene approaches. Over the last two decades, several new families, genera, and species (8, 9, 23) have been proposed based on genome sequence data and phylogenomic analyses. Waite et al. (7) reclassified the order *Myxococcales* into an independent phylum *“Myxococcota”* based on a phylogenetic analysis of 120 single copy conserved marker genes retrieved from genome sequences comprised of seven families, four orders, and two classes. Recently, Chamber et al. (23) and Wang et al. (9) have proposed the merging of genera *Myxococcus* and *Pyxidicoccus* based on genome-related indices such as (i) digital DNA-DNA hybridization, (ii) average nucleotide identity, (iii) average amino acid identity, and (iv) percentage of conserved proteins, as well as phylogeny (based on 16S rRNA and core genes). In this context, one of the primary objectives of the study has been to shed light on the systematics of this group and provide an alternative set of parameters for analyzing genus-level demarcation. In addition, one novel genus and two novel species, *Pseudomyxococcus flavus* gen. nov. sp. nov. and *Myxococcus faecalis* sp. nov., isolated from human fecal samples were described through phenotypic, phylogenomics, and functional approaches.

## RESULTS

### Phenotypic characterization

This section focuses on isolation and characterization of the novel myxobacterial strains from IBD fecal samples. A comprehensive approach was employed, encompassing morphological, physiological, and biochemical analyses. The key finding provides insights into their growth parameters, enzymatic activities, fatty acid profile, predatory behavior, and antibiotic susceptibility. The strains O35 and O15 were closely related to *Myxococcus fulvus* DSM 16525^T^, and strain Y35 to *Myxococcus hansupus* mixupus (described below). Thus, in this study, we analyzed these two reference strains for both physiological and genomic characteristics as per recommendations by Tindall et al. (24) for phenotypic methods and Chun et al. and Riesco and Trujillo (25, 26) for comparative genomic analysis. Furthermore, *M. hansupus* mixupus was isolated and described (genomic characteristics) by our group previously (27), but it was not validly described. In this study, a formal species description of the taxon, along with its genomic characteristics, is provided.

#### Isolation, morphology, and physiology

**Fig 1:**
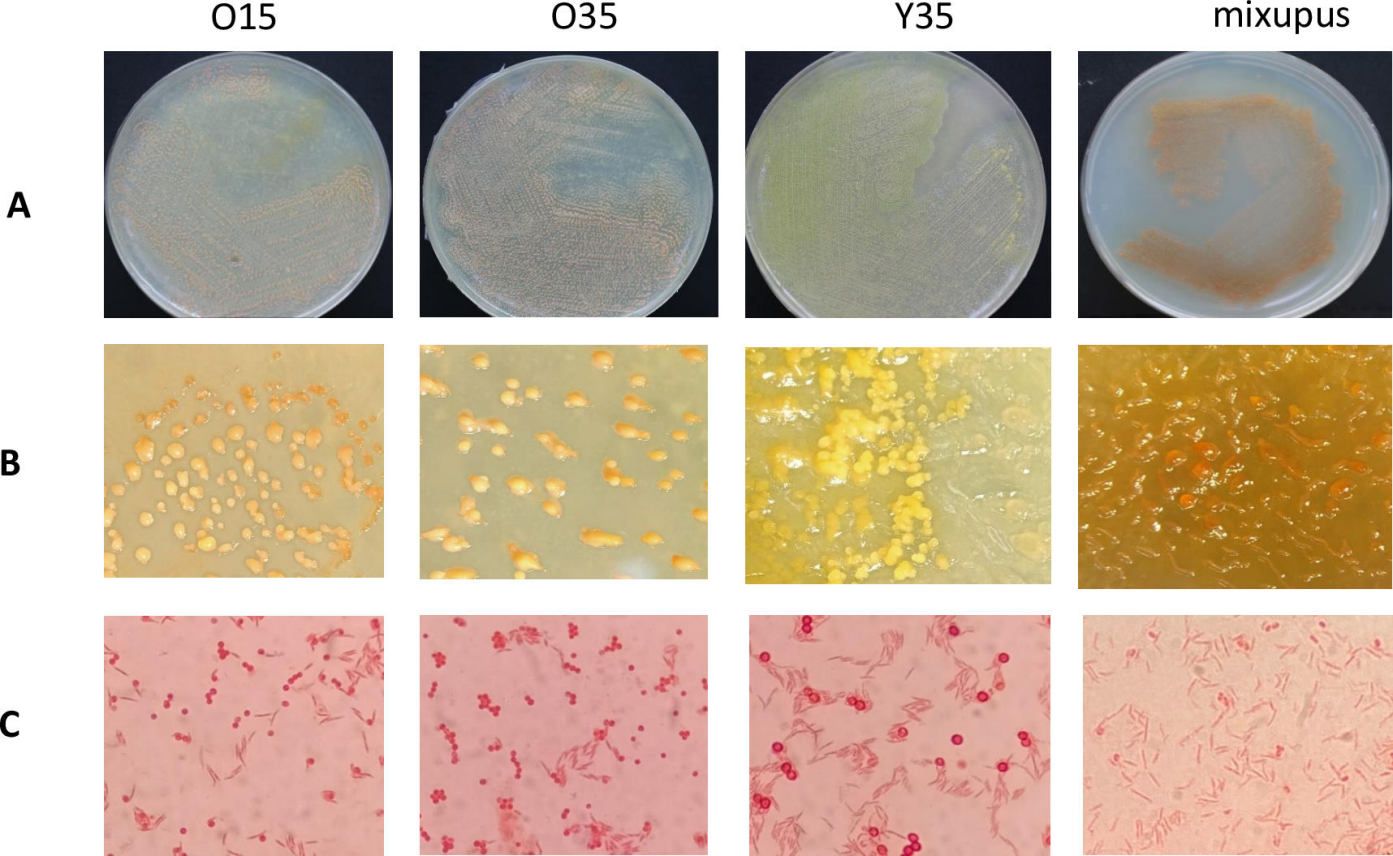
Morphological characteristic of the novel strains. (A) Colonies of *Myxococcus* spp. O15, O35^T^, Y35^T^, and *Myxococcus hansupus* mixupus^T^ on VY/2 plates, (B) stereomicroscopic micrographs of fruiting bodies of *Myxococcus* spp. O15, O35^T^, Y35^T^, and *Myxococcus hansupus* mixupus^T^, (C) gram stained vegetative cells and myxospores of *Myxococcus* spp. O15, O35^T^, Y35^T^, and *Myxococcus hansupus* mixupus^T^.

**Fig 2:**
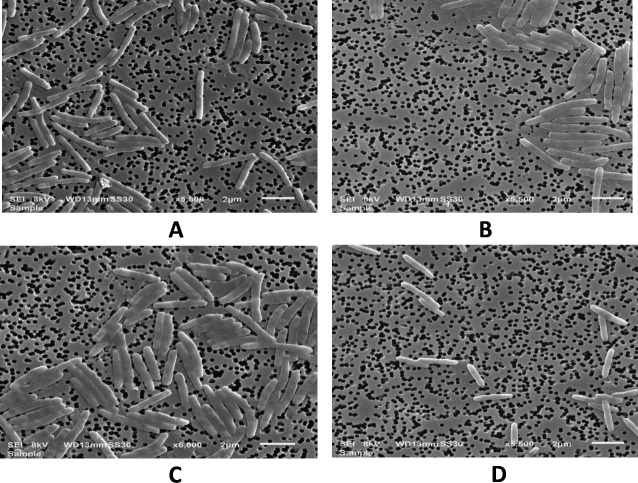
Scanning electron micrographs of the four novel strains. (A) *Myxococcus* sp. O15, (B) *Myxococcus* sp. O35^T^, (C) *Myxococcus hansupus* mixupus^T^ and (D) *Myxococcus* sp. Y35^T^. Bars represent 2 µm.

Three myxobacteria-like strains, O35, O15, and Y35, were isolated from human faeces using the *Escherichia coli-*baiting technique. The strains O35, O15, and *M. hansupus* mixupus formed globular slimy mound-like pink fruiting bodies, and strain Y35 formed yellow fruiting structures without stalks, showing transparent swarms, rod-shaped vegetative cells, and round-shaped myxospores, characteristic features of the genus *Myxococcus* (28) (Fig. 1 and 2; Fig. S1; Table 1). Out of three new isolated strains (now identified as members of the genus, as described below), *Myxococcus* sp. O35, O15, Y35, and *M. hansupus* mixupus grew optimally at pH 9.0 (Table 2). *Myxococcus* sp. O35 and O15 differed from *Myxococcus* sp. Y35 and *M. hansupus* mixupus in growth temperature profile as the former pair showed optimum growth at 30°C–37°C, whereas the latter grew optimally at 28°C–30°C (Table 2). All the strains grew without NaCl; however, optimum growth was recorded at 0.5% (wt/vol) and 1% (wt/vol) NaCl, respectively, for the pairs *Myxococcus* sp. O15-O35 and *Myxococcus* sp. Y35-*M. hansupus* mixupus (Table 2).

**TABLE 1:** Comparative phenotypic characteristics of *Myxococcus* spp. O15, O35^T^, Y35^T^, *M. fulvus* DSM 16525^T^ (Mf)^*c*^, and *Myxococcus hansupus* mixupus^T^ (Mh)^*a*^

Test	O35	O15	Mf	Y35	Mh
Cell size and shape	0.41–0.69 × 2.91–4.56 µm, rod	0.40–0.51 × 2.75–4.64 µm, rod	0.6–0.8 × 4–8 µm, rod^*c*^	0.42–0.46 × 2.39–3.14 µm, rod	0.40–0.49 × 2.74–4.38 µm, rod
Morphology					
Spore size and shape	1.22–1.45 µm, moderate in size, round shape	1.26–1.34 µm, moderate in size, round shape	1.3–1.6 µm, moderate in size, round shape^*c*^	1.27–1.39 µm, moderate in size, round shape	1.08–1.40 µm, moderate in size, round shape
Fruiting body	Soft, slimy, globular mound, orange	Soft, slimy, globular mound, orange	Soft, slimy, globular mound, orange	Soft, slimy, globular mound, yellow	Soft, slimy, globular mound, orange
Swarming colony	Unpigmented, transparent	Unpigmented, transparent	Unpigmented, transparent	Unpigmented, transparent	Unpigmented, transparent
Urease	−	−	−	−	−
Hydrolysis of					
Esculin	+	+	–	−	−
Acid production					
Glucose	−	−	−	+	−
Ribose	−	−	−	+	−
Raffinose	−	−	−	+	−
API 20 NE test^*b*^					
Nitrate reduction	−	−	+	−	−
Urease	−	−	+	−	−
p-Nitrophenyl-beta- D-galactopyranoside	−	+	+	−	+
Assimilation of					
Glucose	−	−	+	−	+
Arabinose	−	−	+	−	+
Mannose	−	+	+	+	+
Mannitol	+	+	+	+	+
N-acetyl- glucosamine	+	+	+	+	+
Maltose	+	+	+	+	+
Gluconate	+	+	+	+	+
Caprate	+	+	−	+	+
Adipate	−	+	−	+	+
Malate	+	+	+	−	+
Citrate	−	+	+	−	−
Phenyl acetate	−	+	−	−	−
API ZYM					
Beta-glucosidase	+	+	−	−	−
Alpha-galactosidase	+	+	−	−	−
α-chymotrypsin	+	+	+	−	−

^*a*^ All strains were catalase positive and oxidase negative. Positive for hydrolysis of esculin (incase of API 20NE, while negative in plate assay for strains Mf, Y35, and Mh), gelatin, casein, tween 20 and 80, and negative for xylan, urea and starch hydrolysis. Acid production from arabinose, galactose, and sucrose was negative in all strains. In API 20 NE kit, tests for indole production, glucose acidification, and arginine dihydrolase were negative, whereas gelatin and esculin hydrolysis were positive in all strains. Assimilation of mannitol, N-acetyl-glucosamine, maltose, gluconate, and caprate were positive in all strains. The enzymatic activity of alkaline phosphatase, esterase (C 4), esterase lipase (C 8), lipase (C 14), leucine arylamidase, valine arylamidase, acid phosphatase, and naphthol-AS-BI-phosphohydrolase were positive in all strains. + indicates positive, and − indicates negative.

^*b*^ Data of API 20 NE for *M. fulvus* DSM 16525^T^ (Mf) adapted from Chambers et al. (23).

^*c*^ Data of cell size and shape for *M. fulvus* DSM 16525^T^ adapted from Reichenbach (28).

**TABLE 2:** Physiological growth characteristics (range of pH, NaCl%, and temperature) of *Myxococcus* spp. O15, O35^T^, and Y35^T^, *M. fulvus* DSM 16525^T^ (Mf) and *M. hansupus* mixupus^T^ (Mh)^*a*^

Characteristics	O35	O15	Mf	Y35	Mh
Growth at different temperatures (pH 7.8 and NaCl% 0)					
25°C	++	++	+++	+	++
28°C	+++	++	+++	++	+++
30°C	+++	+++	+++	+	+++
37°C	+++	+++	+++	+	+
40°C	+	+	−	+	+
Growth at different pH (Nacl% 0, temperature 30°C)					
pH 5.0	−	−	−	−	−
pH 6.0	++	++	++	++	+
pH 7.0	++	++	++	++	++
pH 8.0	++	++	+++	+++	+++
pH 9.0	+++	+++	++	+++	+++
Growth at different NaCl% (pH 7.8, temperature 30°C)					
0.0	+++	+++	+++	+	+
0.5	+++	+++	+++	++	++
1.0	++	++	++	+++	+++
1.5	+	+	+	+	+
2.0	−	−	−	−	−

^*a*^ +++ indicates fast growth, ++ indicates moderate growth, + indicates slow growth, and − indicates no growth.

#### Biochemical tests and cellular fatty acids

With respect to acid production/utilization of substrates, the strains differed in adipate, citrate, glucose, malate, mannose, phenylacetate, raffinose, and ribose (Table 1). In the API ZYM enzymatic profiles, strains showed identical profiles, with the exception of *Myxococcus* sp. Y35 and *M. hansupus* mixupus that were negative for α-chymotrypsin, alpha-galactosidase, and beta-glucosidase (Table 1). The major fatty acids (≥5%) in four strains belonged to branched chain fatty acids, namely anteiso-C_15:0_, iso-C_15:0_, anteiso-C_17:0_, and anteiso-C_14:0_ (except in *Myxococcus* sp. Y35, Table 3). The fatty acids iso-C_15:0_ and C_16:1_ ω5c are biomarkers reported in the genus *Myxococcus* (29); however, in our study, the latter was observed as a minor component (~2%–5%). Moreover, few fatty acids, such as anteiso-C_11:0_, anteiso-C_13:0_, and iso-C_11:0_ 3OH, were uniquely abundant in *Myxococcus* sp. O15 and *Myxococcus* sp. O35-*M. hansupus* mixupus, respectively (Table 3).

**TABLE 3:** Cellular fatty acid profiles of *Myxococcus* sp. O15, O35^T^, Y35^T^, *M. fulvus* DSM 16525^T^ (Mf), and *M. hansupus* mixupus^T^ (Mh)^*a*^

Fatty acid	Mf	O35	O15	Mh	Y35
Straight chain					
C_17:1_ ω7c	–^*b*^	2.78	2.13	5.73	4.57
C_16:0_	1.31	3.91	2.23	4.56	4
C_15:0_ 2OH	–	–	1.79	5.02	3.7
C_20:4_ ω6,9,12,15c	–	2.35	–	–	3.27
C_16:1_ ω5c	1.24	4.82	3.11	1.8	2.03
C_12:0_	–	–	–	–	1.75
C_11:0_ 3OH	–	1.28	–	2.16	1.7
C_14:0_	–	1.49	–	–	1.36
C_20:1_ ω7c	–	–	–	–	1.27
C_15:1_ ω5c	–	–	–	1.32	1.04
C_18:0_	–	1		–	–
C_18:3_ ω6c (6,9,12)	1.05	4.49	3.25	–	–
C_9:0_ 3OH	–	–	1.15	–	–
Branched chain					
anteiso-C_15:0_	**61.89^*c*^**	**11.22**	9.29	**22.1**	**18.54**
iso-C_15:0_	**10.01**	**15.72**	7.05	1.81	**12.36**
anteiso-C_17:0_	1.9	6.39	5.38	**12.59**	**10.8**
iso-C_17:0_	0.51	**11.87**	2.96	1.02	7.51
iso-C_16:0_	1.8	4.32	2.38	4.49	5.42
iso-C_11:0_ 3OH	**–**	6.07	–	**11.4**	3.96
iso-C_15:1_ G	**–**	1.51	1.12	2.65	2.28
iso-C_15:0_ 3OH	–	–	1.11	–	3.64
anteiso-C_11:0_	4.91	–	**21.95**	–	–
anteiso-C_13:0_	4.17	–	**17.44**	–	–
anteiso-C_17:1_ ω9c	–	–	–	1.84	–
anteiso-C_14:0_	**–**	7.92	5.49	**14.11**	–
anteiso-C_16:0_	–	1.46	1.08	1.7	1.3
anteiso-C_12:0_	–	–	2.78	–	–
iso-C_17:1_ ω10c	–	1.83	–	–	–
iso-C_13:0_	–	1.18	–	–	–
anteiso-C_12:0_	–	–	2.78	–	–
Summed feature 4	–	–	–	–	2.32
Summed feature 3	–	1.7	1.15	–	1.72
Summed feature 8	–	–	–	1.5	1.29
Summed feature 5	–	–	–	2.99	–

^*a*^ Values are indicated as percent abundance.

^*b*^ – indicates either not present or minor fatty acid (<1%).

^*c*^ Fatty acids in bold indicate major components (>10%).

#### Predatory activity and antibiotic susceptibility of the strains

All strains showed good predatory activity against *E. coli* MTCC 1652, *Pseudomonas aeruginosa* MTCC 1934^T^, *Shigella boydii* MTCC 11947^T^, and *Staphylococcus aureus* MTCC 1430^T^ (Table 4). However, none of the strains exhibited predatory activity against *Candida albicans* SC5314 and *Bacillus subtilis* MTCC 121^T^ except *M. fulvus* DSM 16525^T^, which predated all cell types. *M. hansupus* mixupus did not predate *Klebsiella pneumoniae* MTCC 661^T^ and *Vibrio cholerae* MTCC 3904 but was positive for *C. albicans* SC5314 (Table 4). All strains were resistant to ampicillin/sublactum, carbenicillin, gentamycin, piperacillin, streptomycin, and ticarcillin/clavulanic acid and sensitive to ceftriaxone, ciprofloxacin, colistin, levofloxacin, polymyxin, and kanamycin (Table 4).

**TABLE 4:** Antibiotic susceptibility and predation assay of *Myxococcus* spp. O15, O35^T^, Y35^T^, *M. fulvus* DSM 16525^T^ (Mf), and *M. hansupus* mixupus^T^ (Mh)^*a*^

Test	O35	O15	Mf	Y35	Mh
Antibiotic susceptibility					
Kanamycin (250 mg/L)	−	−	−	−	−
Streptomycin (50 mg/L)	+	+	+	+	+
NET	−	+	+	−	−
IPM	+	+	−	+	+
CFS	+	−	+	+	+
TOB	−	−	v	−	+
CPZ	+	+	+	+	−
CTR	−	−	v	−	−
PIT	+	+	+	−	−
TI	+	+	+	−	+
CPM	−	−	+	+	−
PB	+	+	+	+	+
Predation assay					
*E. coli*	+	+	+	+	+
*Staphylococcus aureus*	+	+	+	+	+
*Candida albicans*	−	−	+	−	+
*Pseudomonas aeruginosa*	+	+	+	+	+
*Klebsiella pneumoniae*	+	+	+	+	−
*Bacillus subtilis*	−	−	+	−	−
*Shigella boydii*	+	+	+	+	+
*Vibrio cholerae*	+	+	+	+	−

^*a*^ Predation assay: *E. coli*—MTCC 1652, *Staphylococcus aureus*—MTCC1430^T^, *Klebsiella pneumoniae*—MTCC 661^T^, *Bacillus subtilis*—MTCC 121^T^, *Shigella boydii*—MTCC 11947^T^, *Vibrio cholerae*—MTCC3904, MTCC 1934^T^*—Pseudomonas aeruginosa*, *Candida albicans*—SC5314. Activity was checked as per Morgan et al. (30). Antibiotic susceptibility: AK—Amikacin (30 mcg), AT—Aztreonam (30 mcg), NET—Netillin (30 mcg), IPM—Imipenem (10 mcg), CIP—Ciprofloxacin (5 mcg), GEN—Gentamycin (10 mcg), CFS—Cefeoperazone (75/10 mcg), A/S—Ampicillin/Sulbactam (10/10 mcg), TOB—Tobramycin (10 mcg), PI—Piperacillin (100 mcg), CL—Colistin (10 mcg), TCC—Ticarcillin/Clavuanic acid (75/10 mcg), CPZ—Cefeperazone (75 mcg), CTR—Ceftriaxone (30 mcg), LE—Levofloxacin (5 mcg), PIT—Piperacillin/Tazobactam (100/10 mcg), CB—Carbenicillin (100 mcg), TI—Ticaracillin (75 mcg), CPM—Cefepime (30 mcg), PB—Polymyxin-B (300 units). + indicates resistance, − indicates susceptibility, and v indicates variable. All strains showed resistance toward streptomycin, GEN, A/S, PI, TCC, and CB and sensitivity to kanamycin, CL, CIP, LE, and PB.

Overall, in terms of phenotype, *Myxococcus* sp. O35 and O15 were quite similar in their response, whereas *Myxococcus* sp. Y35 and *M. hansupus* mixupus showed strain-specific characteristics, especially in physiological parameters of temperature and sugar substrates utilization/acid production profiles. *Myxococcus* sp. Y35 and *M. hansupus* mixupus also revealed closer patterns in terms of antibiotic resistance profiles compared to the other two strains (Tables 1 to 4).

### Phylogenomics

To understand the evolutionary relationships of the novel strains, molecular phylogenetic analyses based on 16S rRNA gene and whole genome sequence data were completed. The 16S rRNA gene-based phylogeny provides the primary identification and taxonomic placement within the genus. For whole genome sequence analysis, different parameters of overall genome relatedness indices (OGRI) calculated through average amino acid identity (AAI), average nucleotide identity (ANI), percentage of conserved proteins (PoCP), and phylogeny based on conserved/core genes were utilized to confirm the identification at genus and/or species level. During the course of phenotypic and genome analyses of novel strains, the closest reference taxa represented by type strains of *M. fulvus* DSM 16525^T^ and *M. hansupus* mixupus^T^ were selected as per recommendations for descriptions of novel taxa based on phenotype and genome sequence analysis (24–26).

#### Molecular phylogeny based on 16S rRNA gene

**Fig 3:**
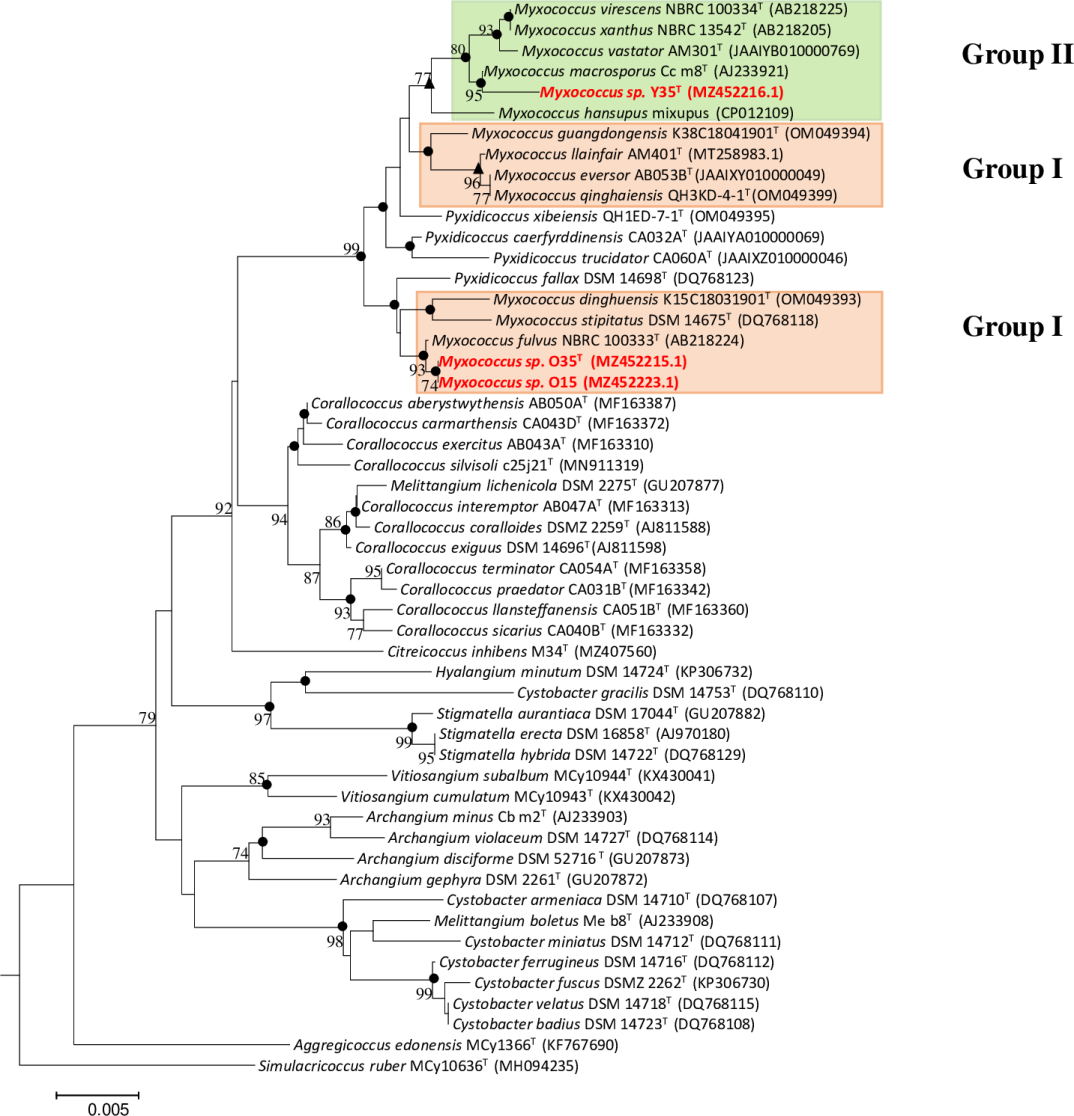
Phylogenetic tree based on 16S rRNA gene sequences. The tree was inferred by the neighbor-joining method using MEGA7 with the kimura-2 parameter model. The tree showing phylogenetic relationship between strains *Myxococcus* sp. O15, O35, Y35, and *Myxococcus hansupus* mixupus and the members of its closely related species of the genera *Myxococcus* and family *Myxococcaceae*. Bootstrap values from 1000 replications and >70% are only shown in branch points. There were a total of 1151 positions in the final data set. Filled circles represents nodes recovered in the maximum-parsimony and maximum-likelihood analysis and filled triangle for maximum-likehood.

A phylogenetic tree based on 16S rRNA gene sequences showed that *Myxococcus* sp. O15 and O35 were closely related to *M. fulvus* DSM 16525^T^ (99.6%–100%), *M. stipitatus* DSM 14675^T^, *M. dinghuensis* K15C18031901^T^, and *Pyxidicoccus fallax* DSM 14698^T^ and grouped in the same clade but distantly related (≤99.0% sequence identity) to other valid species names (Fig. 3). *Myxococcus* sp. Y35 shared the highest sequence similarity with *M. macrosporus* Ccm8^T^ (99.3%), followed by *M. virescens* NBRC 100334^T^ and *M. xanthus* NBRC 13542^T^ (>99%) and showed <99.0% identity to other valid species and was retrieved with *M. macrosporu*s Ccm8^T^ supported by high bootstrap values (Fig. 3). The taxon *M. hansupus* mixupus originally described by our group (27) was further considered in this study as its name has no valid standing in nomenclature yet and was found to have the highest identity with *M. macrosporus* Ccm8^T^ (99.4%) followed by *M. virescens* NBRC 13542^T^ (99.3%) and *M. xanthus* NBRC 13542^T^ (>99.2%) and in the tree was always retrieved as an independent lineage part of a bigger clade consisting of *M. macrosporus* Ccm8^T^, *Myxococcus* sp. Y35, *M. virescens* NBRC 100334^T^, *M. xanthus* NBRC 13542^T^, and *M. vastator* AM30^T^. In the tree, the genus *Myxococcus* was divided into two groups, group I consisting of novel strains *Myxococcus* sp. O15, O35, *M. fulvus* DSM 16525^T^ (type species of the genus), *M. stipitatus* DSM 14675^T^, *M. dinghuensis* K15C18031901^T^, and group II consisted of *Myxococcus* sp. Y35, *M. hansupus* mixupus, *M. virescens* NBRC 100334^T^, *M. xanthus* NBRC 13542^T^, *M. macrosporus* Ccm8^T^, and *M. vastator* AM301^T^. The four recently described species *M. guangdongensis* K38C18041901^T^, *M. qinghaiensis* QH3KD-4-1^T^, *M. eversor* AB053B^T^, and *M. llanfair* AM401^T^ were retrieved separately with the genus *Pyxidicoccus* interspersed between them and group I taxa (Fig. 3). All three tree-making methods, i.e., neighbor joining, maximum parsimony, and maximum likelihood (ML), revealed identical branching patterns for *Myxococcus* sp. O15, O35, Y35, and *M. hansupus* mixupus supported by very high bootstrap values (Fig. 3).

#### Molecular phylogenetic analyses based on whole genome sequences

**Fig 4:**
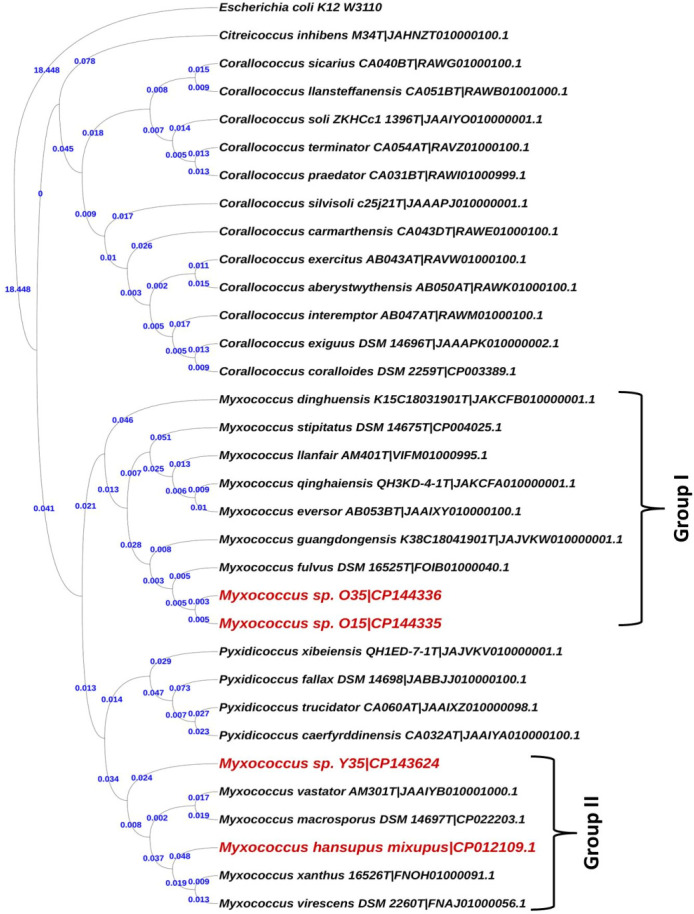
Phylogenetic tree depicting evolutionary relationships of novel strains *Myxococcus* spp. O35^T^, O15, Y35^T^, and *Myxococcus hansupus* mixupus^T^ based on 35 universal single copy genes specific for myxobacteria. The tree was generated using the GTR-GAMMA model of the ML method in RAxML tool and visualized in iTOL.

**Fig 5:**
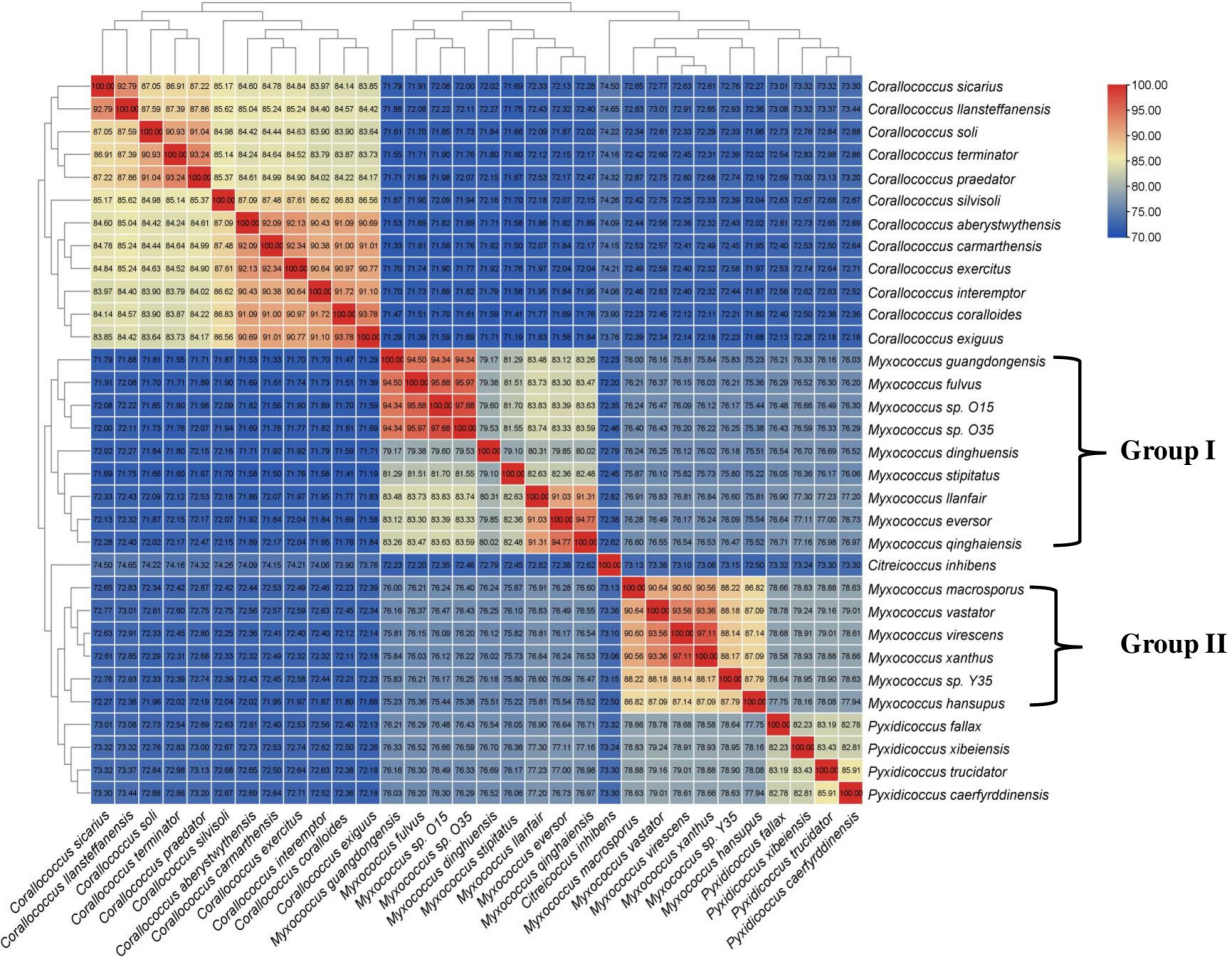
Average amino acid identities (AAI) from pairwise whole genome comparisons. The values of AAI between the genomes are depicted as heatmap along with the tree cladogram.

**Fig 6:**
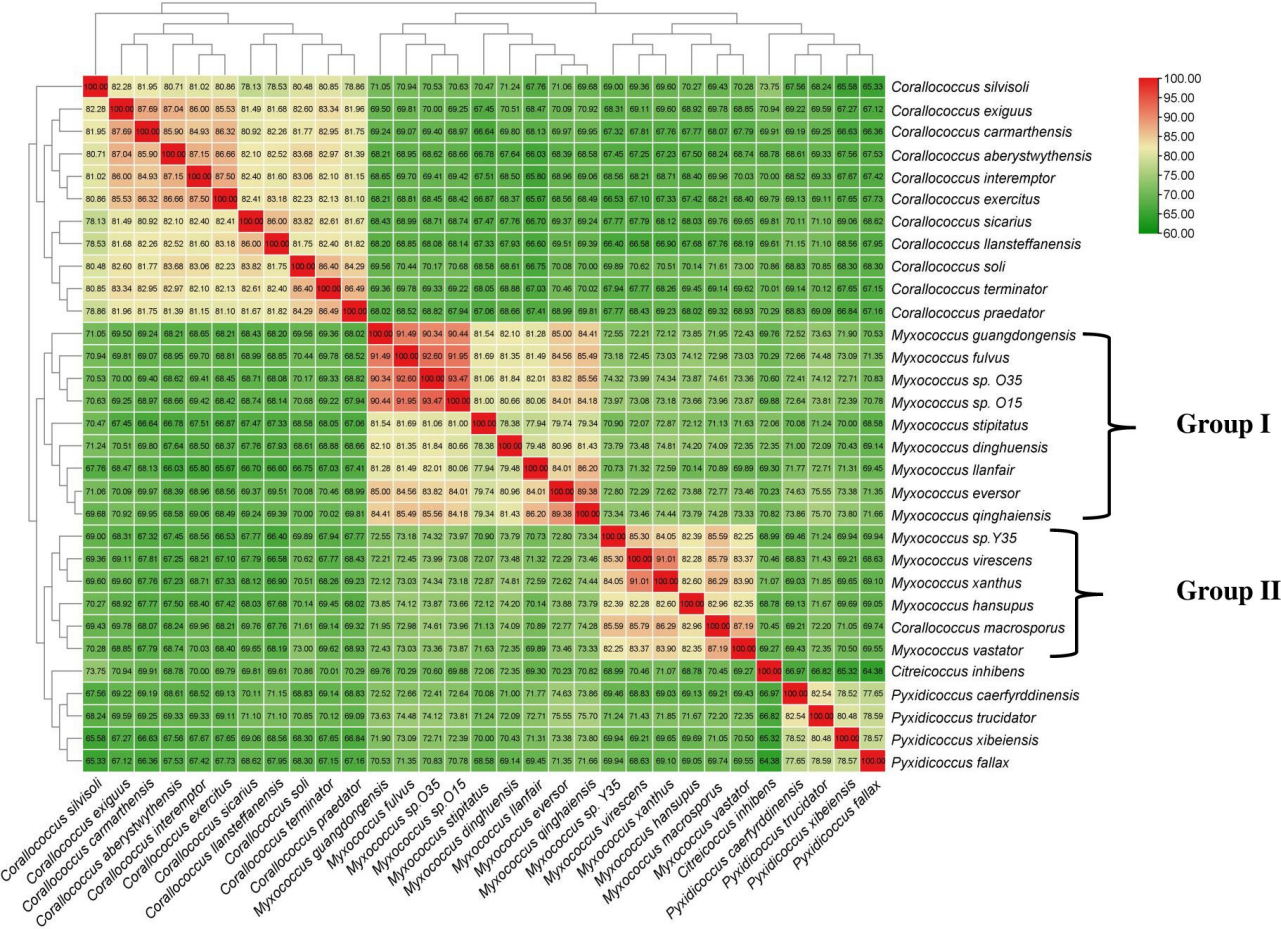
Percentage of conserved proteins (PoCP) from pairwise whole genome comparisons. The values of PoCP between the genomes are depicted as heatmap along with the tree cladogram.

To overcome the limitations of 16S rRNA-based phylogeny and to clearly understand the relationships within the genus *Myxococcus*, trees based on 400 conserved single-copy proteins (using the PhyloPhlan pipeline), 92 core genes (using the UBCG: up-to-date bacterial core gene pipeline), 35 concatenated single-copy marker genes customized for myxobacteria, and RpoC protein were constructed that revealed similar branching as observed in the 16S rRNA-based tree with much better resolution and demarcation of lineages (Fig. 4; Fig. S2 to S4). In these four trees, although *Myxococcus* sp. O15 and O35 still clustered with *M. fulvus* DSM 16525^T^, *M. stipitatus* DSM 14675^T^, and *M. dinghuensis* K15C18031901^T^, the group I also included the four species *M. guangdongensis* K38C18041901^T^, *M. qinghaiensis* QH3KD-4-1^T^, *M. eversor* AB053B^T^, and *M. llanfair* AM401^T^ (that were present in an independent lineage in 16S rRNA based tree [Fig. 3]), supported by high bootstrap values (Fig. 4; Fig. S2 to S4). *Myxococcus* sp. Y35 clustered with *M. macrosporus* Ccm8^T^, *M. hansupus* mixupus^T^, *M. vastator* AM301^T^, *M. virescens* DSM 2260^T^, and *M. xanthus* DSM 16526^T^ as group II Myxococci supported by high bootstrap values similar to the branching pattern in the 16S rRNA tree. One interesting observation was the confirmation of the polyphyletic nature of the genus *Myxococcus* via 16S rRNA gene, RpoC protein, and genome-based trees (UBCG, core 35 and 92 genes and PhyloPhlan) wherein *Myxococcus* sp. O15-O35 and *Myxococcus* sp. Y35-*M. hansupus* mixupus were affiliated to two different groups of *Myxococcus* spp. The heat map dendrogram based on whole proteome genus delimiting similarity indices, i.e., AAI and PoCP, reinforced the above observations and demarcated two groups within the genus *Myxoccoccus* (Fig. 5 and 6). Cluster of orthologous genes (COG)-based functional characterization of *Myxococcus* spp. proteomes majorly supported a similar cladding pattern within the genus *Myxococcus* (Fig. S5).

It was, therefore, clear from the 16S rRNA/genome-based phylogeny and AAI/PoCP similarity indices that the genus *Myxococcus* is polyphyletic and is represented by two groups. Furthermore, the novel strains *Myxococcus* sp. O15-O35 and *Myxococcus* sp. Y35 were retrieved in two separate groups, with *M. fulvus* as the closest species for the former pair. In the case of group II myxococci, *M. hansupus* mixupus was always retrieved stably in the cluster. This strain was described previously by our group but has no valid standing in nomenclature. Therefore, in the course of phenotypic and genome sequence analysis of the three novel strains, *M. hansupus* mixupus^T^ was included for comparative characterization as per standard recommendations for the description of novel taxa (24–26).

#### Calculation and analyses of OGRI (GGDC, ANI, AAI, PoCP)

*Myxococcus* sp. O35, O15, and Y35 shared ≤70% digital DNA-DNA hybridization values (dDDH) calculated through the genome-to-genome distance calculator (GGDC) and ≤95%–96% ANI values with existing valid species names and among themselves (Table S1). This led to the inference that they represent two separate novel species within the genus *Myxococcus* according to gold standard criteria for demarcating bacterial species based on these two genome similarity indices (30–34). *Myxococcus* sp. O35 and O15 shared high but not identical GGDC (78%) and ANI (97%) values, indicating that they belonged to the same species but displayed intraspecies strain variability (Table S1). The genome summary statistics of *Myxococcus* sp. O35, O15, Y35, and *M. hansupus* mixupus with closest reference taxa are listed in Table S2. The genome length varied between ~8.9 (*M. vastator*) and 12.4 Mbp (*M. llanfair*), with genome GC mol% content between 68.7% and 70.7%. The strain pairs *Myxococcus* sp. O15-O35 and *Myxococcus* sp. Y35-*M. hansupus* mixupus differed, respectively, from their closest reference taxa *M. fulvus* DSM 16525^T^ and *M. macrosporus* Ccm8^T^ in size, coding sequences, and total numbers of tRNAs and rRNAs (Table S2), thus highlighting differences in genome characteristics supporting the novel species status. There were also differences between *Myxococcus* sp. O15 and O35 in terms of the above properties, indicating strain-specific characteristics as they were isolated from different patients. Therefore, based on evidence from the above analyses we propose two novel species, *M. faecalis* O35^T^, O15, and *M. flavus* Y35, that belong to two different groups within the genus *Myxococcus* and also formally describe *M. hansupus* mixupus^T^, as this is not a validly described species name until now. The detailed characteristics are given in the species description.

### Functional annotation of genomes

#### Biosynthetic gene clusters

**Fig 7:**
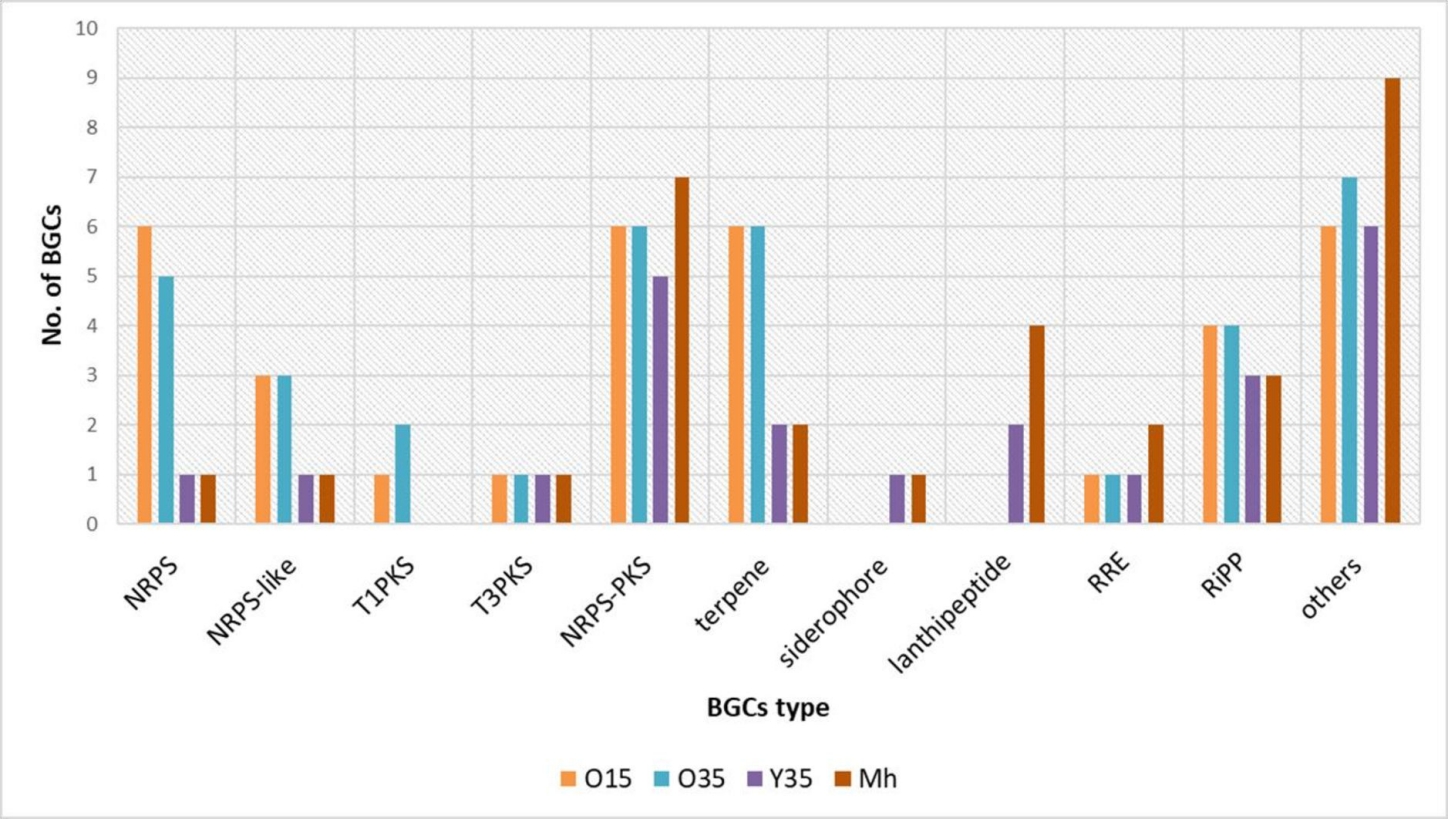
Biosynthetic gene clusters distribution among *Myxococcus* spp. O15, O35^T^, Y35^T^, and *Myxococcus hansupus* mixupus^T^. NRPS, non-ribosomal peptide synthetase; PKS, polyketide synthase; RiPP-like, Other unspecified ribosomally synthesised and post-translationally modified peptide product; RRE, RiPP recognition element; Mh, *Myxococcus hansupus* mixupus^T^.

**Fig 8:**
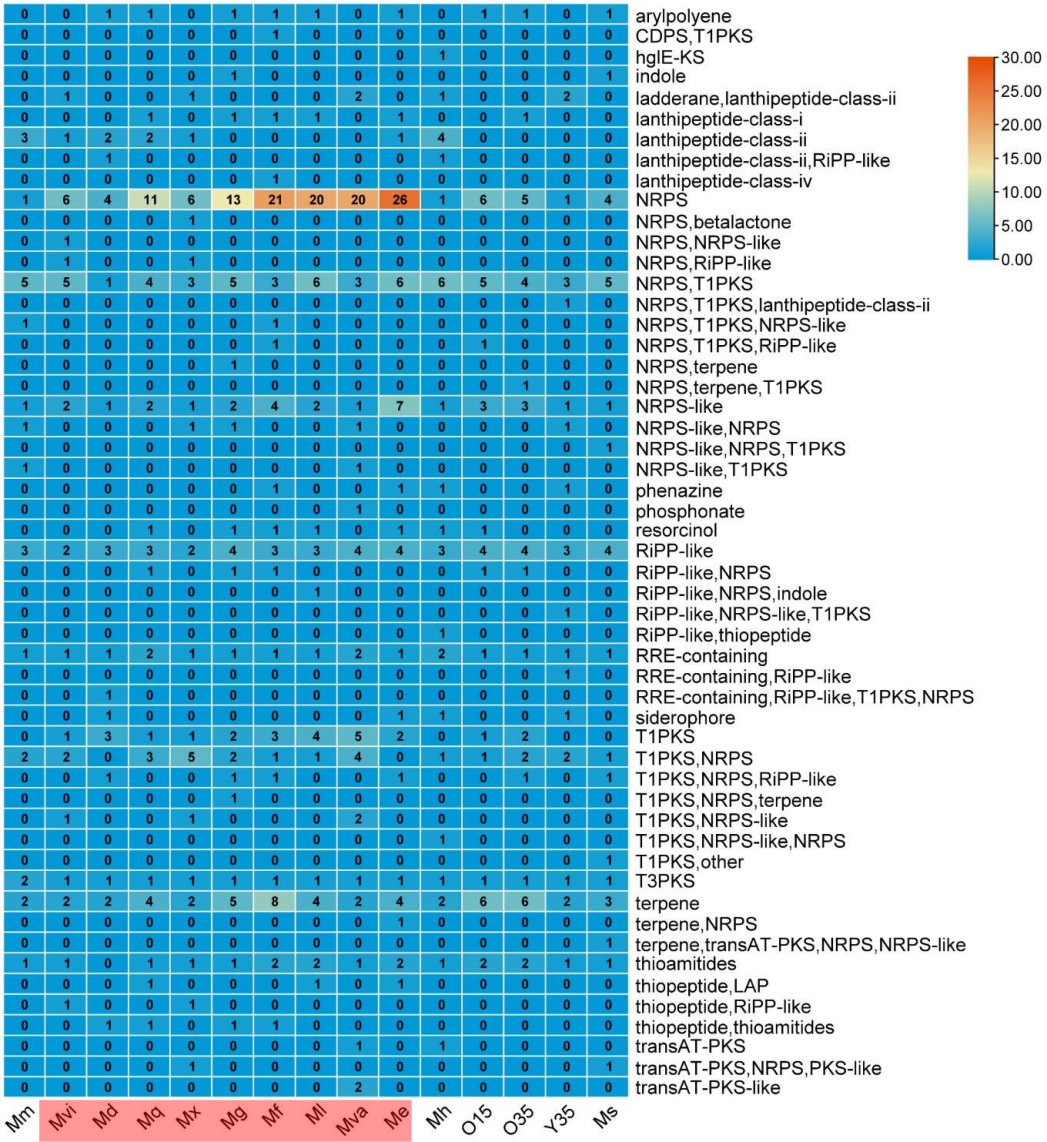
Biosynthetic gene clusters distributions among 15 strains of *Myxococcus* spp. Mx, *Myxococcus xanthus* DSM 16526^T^; Mva, *Myxococcus vastator* AM301^T^; Mm, *Myxococcus macrosporus* DSM 14697^T^; Mh, *Myxococcus hansupus* mixupus^T^; Mvi, *Myxococcus virescens* DSM 2260^T^; Mg, *Myxococcus guangdogensis* K38C18041901^T^; Mf, *Myxococcus fulvus* DSM 16525^T^; Ml, *Myxococcus llanfair* AM401^T^; Ms, *Myxococcus stipitatus* DSM 14675^T^; Me, *Myxococcus eversor* AB053B^T^; Md, *Myxococcus dinghuensis* K15C18031901^T^; Mq, *Myxococcus quinghaiensis* QH3KD-4-1^T^. The strains shown in the red box are the draft genomes.

Myxobacteria are prolific producers of biosynthetic secondary metabolites that play a significant role in predatory behavior (34, 35). These metabolites are also very interesting from a therapeutic point of view due to the antimicrobial/anticancer activity of certain compounds. A total of 567 biosynthetic gene clusters (BGCs) were predicted by AntiSMASH in 15 *Myxococcus* spp. ranging between 23 and 62 (Table S3; Fig. 7 and 8), corresponding to 2.3–6.2 BGCs per 1 Mb (Table S3), and on average, each genome possessed 37 BGCs. *M. eversor* AB053B^T^ contained the highest number of BGCs (36), while *Myxococcus* sp. Y35 contained the lowest (23; Table S3; Fig. 7 and 8). BGCs for non-ribosomal peptide synthase (NRPS) [1-26], NRPS-like [1-7], NRPS-type 1 polyketide synthase (T1PKS) [1-6], ribosomally synthesized and post-translationally modified peptides (RiPP) like [2-4], RiPP recognition element containing [1-2], T3PKS [1-2], T1PKS [1-5], and terpene [2-8] were the most prevalent and majorly present among all *Myxococcus* spp. (Fig. 8).

#### Carbohydrate active enzymes

**Fig 9:**
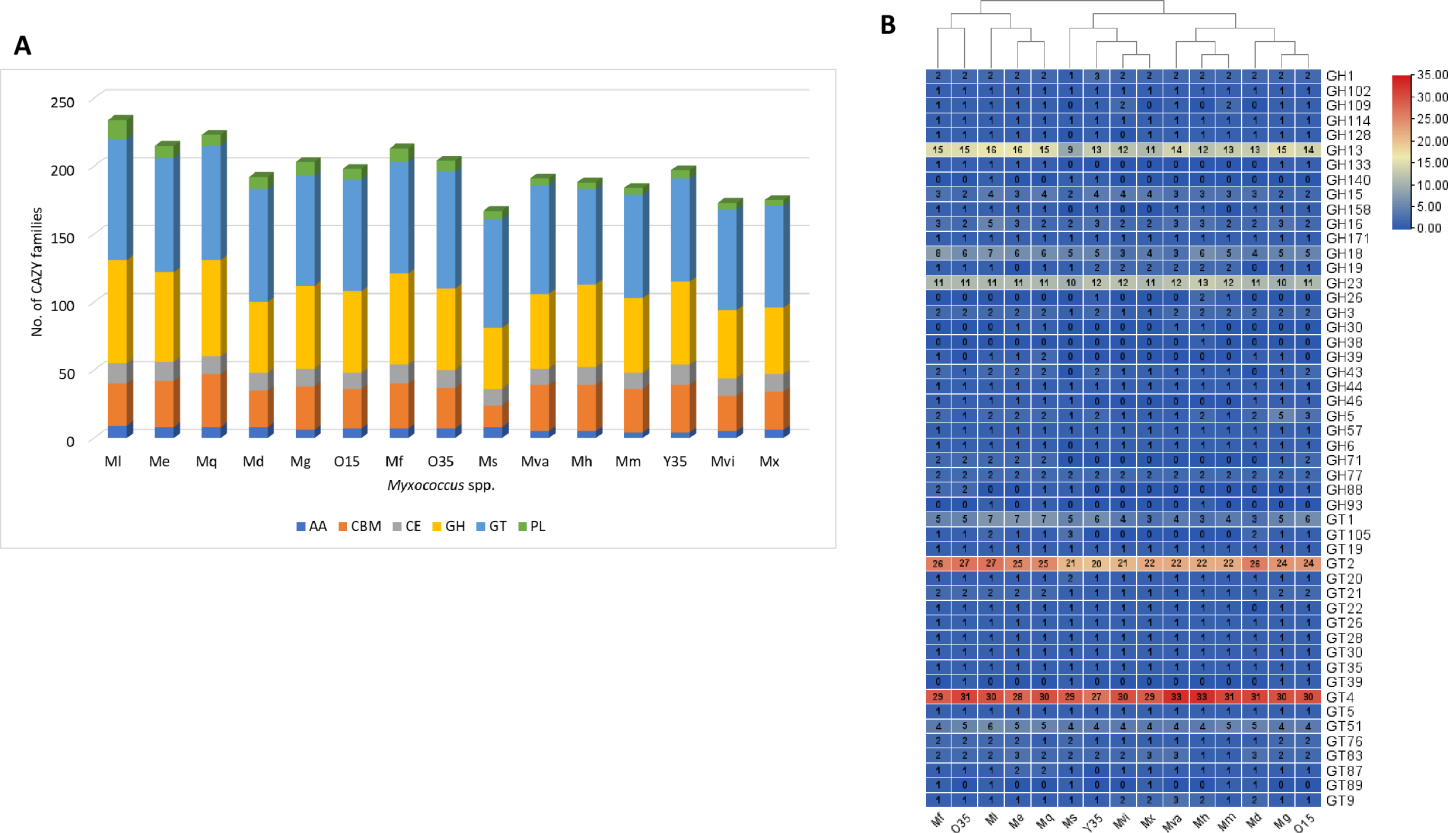
(A) Distribution of CAZymes. GH, glycosyl hydrolase; GT, glycosyl transferase; PL, polysaccharide lyase; CE, carbohydrate esterase; AA, auxiliary activities; CBM, carbohydrate binding module. (B) GH and GT families among 15 strains of *Myxococcus* spp. Mx, *Myxococcus xanthus* DSM 16526^T^; Mva, *Myxococcus vastator* AM301^T^; Mm, *Myxococcus macrosporus* DSM 14697^T^; Mh, *Myxococcus hansupus* mixupus^T^; Mvi, *Myxococcus virescens* DSM 2260^T^; Mg, *Myxococcus guangdogensis* K38C18041901^T^; Mf, *Myxococcus fulvus* DSM 16525^T^; Ml, *Myxococcus llanfair* AM401^T^; Ms, *Myxococcus stipitatus* DSM 14675^T^; Me, *Myxococcus eversor* AB053B^T^; Md, *Myxococcus dinghuensis* K15C18031901^T^; Mq, *Myxococcus quinghaiensis* QH3KD-4-1^T^.

With respect to carbohydrate active enzymes (CAZymes), all *Myxococcus* spp. contained ~200 domains on an average (Fig. 9A; Table S3). The domains involved in degradation and metabolism consisted of glycoside hydrolases (GH), glycosyl transferases (GT), polysaccharide lyases (PL), carbohydrate esterases (CE), and carbohydrate-binding modules (CBM) that represent a diverse set of CAZy families. In addition, the auxiliary activity (AA) family contained mainly enzymes involved in various redox transformations (37–39) (Fig. 9A). The CBM domains enhance the catalytic activity of the appended CAZy family, but they do not exhibit catalytic activity on their own (40). A comparative analysis identified a large number of GT2, GT4, GH13, GH23, GH18 domains and at least one GH16, GH3, GH15, GH1, GH77, GH44, GH5, GH57, and GT1, GT9, GT20, GT21, GT26, GT28, GT30, GT35, GT5, GT51, GT76, GT83, GT87, and GT9 family proteins in the *Myxococcus* spp. (Fig. 9B). A maximum of 234 CAZymes were encoded in the *M. llanfair* AM401^T^ genome, whereas the fewest (167) were detected in *M. stipitatus* DSM 14675^T^ (Fig. 9A; Table S3). All the *Myxococcus* spp. contained at least one gene from cellulose-degrading endoglucanases GH5, GH6, GH44, and β-glucosidases of GH1 and GH3 families, wherein GH6 was present in all except *M. stipitatus* DSM 14675^T^. Starch-degrading amylases/glucoamylases (GH13, GH57, GH77, and GH15), pullulanase/β-1,6-glucosidase (GH13, GH77, and GH57), and chitin-degrading hydrolase enzymes (GH18 and GH19) were abundant in the novel species (Fig. 9B; Table S4). GH-family genes also act in defence and predation such as lysozyme, endolysins, and chitinases (37, 41). These included the GH16, GH18, GH19, and GH23 domains predominantly present across all *Myxococcus* spp. with the exception of *M. dinghuensis* K15C18031901^T^ and *M. eversor* AB053B^T^ whose genomes did not encode GH19. BLASTp analysis of GH16 identified laminarinase β-1,3-glucanase in *M. faecalis* O35, O15, and *M. flavus* Y35 (Table S4) that degraded laminarin, an algal and fungal cell wall component, in novel species *M. faecalis* O15 and *M. flavus* Y35, whereas, both GH18 and GH19 were annotated as chitinases. GH23 a lytic transglycosyalse catalyses the non-hydrolytic cleavage of bacterial cell wall peptidoglycan structures.

It was interesting to note that the heat map based on the CAZyme profile was somewhat similar to the branching pattern obtained from gene-based and genome-based phylogenies. One group consisted of *M. llanfair* AM401^T^, *M. eversor* AB053B^T^, *M. qinghaiensis* QH3KD-4-1^T^, *M. dinghuensis* K15C18031901^T^, *M. guangdongensis* K38C18041901^T^, *M. fulvus* DSM 16525^T^ and the novel proposed species *M. faecalis* O35, O15; and the second cluster consisted of *M. vastator* AM30^T^, *M. hansupus* mixupus, *M. macrosporus* Ccm8^T^, *M. virescens* DSM 2260^T^, *M. xanthus* DSM 16526^T^, and the novel species *M. flavus* Y35^T^ (Fig. 9). The only difference was *M. stipitatus* DSM 14675^T^, retrieved in a separate independent lineage. This aspect has not been addressed previously and can give clues related to evolutionary relationships within the order *Myxococcales*.

#### Gut myxobacterial OTUs

To confirm whether myxobacteria are actually residents of the human gut, 16S rRNA gene-based meta-barcoding data from 51 IBD patient samples (accession no. PRJNA1204524) were analyzed to identify myxobacterial OTUs. About 11 myxobacterial OTUs were detected from different individuals, wherein 10 were from active ulcerative colitis (UC) cases (OTU 245, OTU 122, OTU 201, OTU 131, OTU 335, OTU 369, OTU 394, OTU 435, OTU 39, OTU 384, and OTU 475, Fig. S6) and one was retrieved from the control sample (OTU 475, Fig. S6). The OTUs belonged to the families *Myxococcaceae*, *Polyangiaceae,* and *Nannocystaceae*. The strains *M. faecalis* O35^T^ and O15 were closely related to OTU 131 (IMT 80) retrieved from a biopsy sample of a 45-year-old female patient different from the source of isolation of two strains (Fig. S6). *M. flavus* Y35^T^ was closely related to OTU245 IMS1A obtained from a stool sample of a 32-year-old male. The remaining OTUs were distributed among the genera *Cystobacter, Myxococcus,* and *Pseudenhygromyxa*. Furthermore, in order to determine the sampling environments of the closest metagenomes for *M. faecalis* O35^T^, O15, and *P. flavus* Y35^T^ (i.e., metagenomes containing a query genome), we also searched these genomes against metagenome data in the sequence read archive (SRA). However, none of the three genomes were detected in human-associated metagenome samples. The top three sampling environments based on the count of metagenomes identified for the three genomes were soil, rhizosphere, and root (Table S5).

## DISCUSSION

Among total ~1,034 *Myxococcus* strains reported in the BacDive database (as of January 2024), 37% were from environmental samples (majority were from soil), 38% from hosts (plant + mammals), 15% from host body product (e.g., herbivores dung), 8% from host body site, and 1% from others (Fig. S7). It is evident that the association of myxobacteria has been poorly reported in the context of human hosts (Fig. S7). Myxobacterial OTUs were detected through 16S rRNA gene-based tag sequencing analyses in our own research of IBD patient samples (both biopsy and fecal, Fig. S6 and discussed in a separate section below) and a previous report of one CD patient (14). To further understand the functional roles of bacterial populations and their interactions with the human gut, isolation of pure cultures and genome-wide association studies are necessary. Taking this perspective into consideration, isolation of myxobacteria from human faeces with detailed phenotypic and genome sequence-based functional characterization of three strains belonging to two novel *Myxococcus* spp was undertaken.

### Polyphyly within genus *Myxococcus* and proposal of a novel genus *Pseudomyxococcus*

The taxonomic placement of genera *Myxococcus* and *Pyxidicoccus* is currently not clear, and there is a debate if both these taxa should be merged into a single genus (9, 23). In earlier reports (7, 9, 23), the genus *Myxococcus* was split into two major groups: one group contained seven species, including *M. fulvus*, while the other group harbored five species, including *M. xanthus* as obtained in earlier analysis. Phylogenetic analysis revealed that group II was found to contain distantly related clades with the genera *Pyxidicoccus* and *Citreicoccus* (9). In the present study, three novel myxobacterial strains identified as *M. faecalis* O35^T^, O15, and *M. flavus* Y35^T^ were closely related to *M. fulvus* DSM 16525^T^ and *M. hansupus* mixupus^T^, respectively, based on 16S rRNA gene sequence analysis. Since the latter species name is not validly described, therefore, its taxonomic position was further investigated (27). As it’s a known fact that phylogeny solely based on a single marker gene (e.g., the 16S rRNA gene) cannot resolve species/genus-level affiliation in slowly-evolving taxa such as myxobacteria (8, 23, 42–44), therefore, phylogenetic analysis based on whole genome, other marker genes, and whole proteome-based parameters were taken into consideration. Trees based on RpoC protein, 400 conserved proteins (PhyloPhlan), UBCG core genes, and 35 concatenated single-copy marker genes revealed the polyphyletic nature of the genus *Myxococcus. M. faecalis* O35^T^, O15 clustered with the type species *M. fulvus* DSM 16525^T^, *M. stipitatus* DSM 14675^T^, *M. dinghuensis* K15C18031901^T^, *M. guangdongensis* K38C18041901^T^, *M. qinghaiensis* QH3KD-4-1^T^, *M. eversor* AB053B^T^, and *M. llanfair* AM401^T^ (group I; Fig. 4; Fig. S2 to S4). *M. flavus* Y35^T^ and *M. hansupus* mixupus^T^ were obtained in association with *M. macrosporus* Ccm8^T^, *M. virescens* DSM 2260^T^, *M. xanthus* DSM 16526^T^, and *M. vastator* AM30^T^ (group II). Although *M. faecalis* O35^T^ showed close respective values of GGDC and ANI of ~62% and 95.5% with *M. fulvus* DSM 16525^T^, it differed in phenotypic properties such as esculin hydrolysis, nitrate reduction, adipate, glucose, arabinose, caprate, citrate, and mannose assimilation, predation assay for *C. albicans* SC5314 and *B. subtilis* MTCC 121^T^ (Tables 1 and 4) that supported its novel species status. In terms of genome-based GGDC and ANI values, *M. flavus* Y35^T^ showed low relatedness,,, in terms of both GGDC and ANI values with *Myxococcus* spp. of group II (Table S1), thereby justifying its identification as a novel species. *M. flavus* Y35^T^ differed from *M. hansupus* mixupus^T^ in p-nitrophenyl-β-D-galactopyranoside hydrolysis, glucose, arabinose, and malate assimilation, acid production from glucose, raffinose and ribose, fatty acid profile, predation assay against *C. albicans* SC5314, *K. pneumonia* MTCC 661^T^ and *V. cholera* MTCC 3904^T^, optimum growth temperatures, and pH (Tables 1 to 4).

In this study, we evaluated the usefulness of genus delimiting genome-based similarity indices, i.e., AAI and PoCP (45–50) for the family *Myxococcaceae* by determining threshold values of these two parameters through heat map clustering among different genera referred to as clusters (Fig. 5 and 6). In terms of intercluster AAI values, they were in the range of 71.1–79.2 (Fig. 5; Table S6), i.e., less than ≤80%. The intracluster values ranged from 80.0 to 97.6 (Fig. 5), except for *M. dinghuensis*, which was marginally close to 80.0% but more or less always ≥80. Similarly, inter- and intra-cluster PoCP values ranged from 64.3% to 74.8% (with the exception of 75.7% for the pair *M. qinghaiensis–P. trucidator*) and 77.6%–93.4%, respectively (Fig. 6; Table S6). With respect to PoCP, the threshold values seemed to be about 75% for separating members of different clusters (Table S6; Fig. 6). The intercluster AAI values were below the threshold level (80%) recommended for demarcating genera in other bacterial groups (47, 51). Chamber et al. (23) observed that *P. fallax* DSM 14698^T^ was as distinct from *Myxococcus* spp. as the *Myxococcus* spp. are distinct from each other based on their 16S rRNA gene sequences, DDH/ANI parameters, and pan/core genomes. However, *Corallococcus* spp. are much more distinct than any pair of *Myxococcus*/*Pyxidicoccus* spp. Similarly, Wang et al. (9) had suggested the merging of genera *Myxococcus* and *Pyxidicoccus* based on parameters of AAI and PoCP. In fact, Chamber et al. (23) went a step further and recommended merging the genera *Corallococcus*, *Myxococcus*, and *Pyxidicoccus* based on the criteria of threshold ANI values of 75% determined for actinobacterial taxa *Rhodococcus* (52). It is to be noted that the universal PoCP cut-off boundary of 50% initially proposed by Qin et al. (46) could not be applied to our species groups (clusters) and to several other different bacterial groups, i.e., *Roseobacters, Pseudomonas*, *Bacillus*, *Geobacillus*, *Rhizobeaceae*, *Desulfovibrionaceae,* etc. (48–50, 53–58). Furthermore, the members of the genera *Corallococcus*, *Myxococcus*, and *Pyxidicoccus* had quite different fruiting body morphologies. Members of *Pyxidicoccus* formed hard fruiting bodies, arranged in clusters and consisting of sporangioles, whereas in the other two genera, the fruiting body didn’t contain any sporangioles. *Myxococcus* spp. form hump-, knob-, mound-like structures while *Corallococcus* spp. formed coral, hornlike structures (59). Thus, in contravention to earlier findings, it is concluded that myxobacterial species within each of the above clusters, i.e., groups I and II Myxococci, *Pyxidicoccus*, *Citreicoccus*, and *Corallococcus* were distinct and required reclassification into separate genera. It is thus proposed to retain the name *Myxococcus* for group I myxococci containing type species *M. fulvus* DSM 16525^T^, novel species *M. faecalis* O35^T^, and six species *M. stipitatus* DSM 14675^T^, *M. dinghuensis* K15C18031901^T^, *M. guangdongensis* K38C18041901^T^, *M. qinghaiensis* QH3KD-4-1^T^, *M. eversor* AB053B^T^, and *M. llanfair* AM401^T^. A new genus *Pseudomyxococcus* is proposed for novel species *P. flavus* Y35^T^, and five members of group II myxococci are reclassified as *P. hansupus* mixupus^T^ comb. nov., *P. macrosporus* Ccm8^T^ comb. nov., *P. virescens* DSM 2260^T^ comb. nov., *P. xanthus* DSM 16526^T^ comb. nov., *P. vastator* AM30^T^ comb. nov., and *P. virescens* comb. nov. DSM 2260^T^. Within this new genus *P. virescens* comb. nov. DSM 2260^T^ has been assigned as type species as the oldest name gets priority to be designated as type species.

### Functional potential of the *Myxococcus* spp.

Myxobacteria might influence their ecological niche and gut microbial ecosystem through the secretion of secondary metabolites and carbohydrate-degrading enzymes. The section provides a comparative view of the metabolic potential and functional capabilities of the novel strains with other members of the genus by analysing their BGCs and CAZyme profiles.

#### Diversity and distribution of BGCs

About 567 putative BGCs were predicted by antiSMASH in 15 *Myxococcus* spp. (Table S3). *M. faecalis* O35^T^, O15, *P. flavus* Y35^T^, *P. hansupus* mixupus^T^ and other taxa displayed a wide range of BGCs, ranging from 23 to 58 (Table S3; Fig. 7; Fig. S8 and S9). Differences in the distribution of BGCs were apparent in few group I Myxococci (*M. fulvus* DSM 16525^T^, *M. guangdongensis* K38C18041901^T^, *M. qinghaiensis* QH3KD-4-1^T^, *M. eversor* AB053B^T^, and *M. llanfair* AM401^T^) containing higher numbers of BGCs compared to group II species (except *P. vastator* AM30^T^) (Fig. 8; Table S3). Since the majority of the *Myxococcus* spp. genomes (9 out of 15) were drafts containing several contigs, it might be possible that the number of BGCs was over-predicted in those species. A high number of unknown BGCs were present in *Myxococcus* spp. genome. Out of 567, the majority showed low/no homology (132 showed <50% sequence similarity and 280 revealed no hits) with the MIBIG database (Fig. S9A). The analysis suggested that genomes of myxobacteria encoded several NRPS, PKS, NRPS-PKS hybrids, and lantipeptide gene clusters which might assist in competition and predation. The known core metabolites identified in all *Myxococcus* spp. belonged to geosmin, VEPE/AEPE/TG-1, carotenoid, and myxoprincomide-c506 that demonstrated effective antibacterial/antifungal properties and played a vital role in multicellular development, defence, and predation (9, 23, 60) (Fig. S9B). The VEPE/A-EPE/TG-1 cluster coded for unusual iso-branched vinyl and alkyl ether lipids important for fruiting body formation and sporulation (61). Geosmin, a terpene-type BGC, and myxoprincomide c-506, a peptide antibiotic, have respective functional roles in the defense and predation of *B. subtilis* and *E. coli* cells (36, 62). The carotenoid pigments protect cells from highly reactive singlet oxygen species (63, 64). The other abundant secondary metabolites observed in the majority of *Myxococcus* spp. were myxochelin A/myxochelin B (except for *M. hansupus* mixupus^T^ and *M. stipitatus* DSM 14675^T^), and Dkxanthene both synthesized from NRPS/NRPS-hybrid BGCs, respectively, with antitumor, antiproliferative, anti-bacterial activities against few taxa (65) and a unique class of polyene pigment responsible for yellow color that acts as a pheromone for myxospore formation (66). Among the strains analyzed, Dkxanthene was absent in *P. vastator* AM30^T^*, M. llainfair* AM401^T^*, M. faecalis* O15, and *M. quinghaiensis* QH3KD-4-1^T^ (Fig. S9B). Few strain-specific compounds were detected among *Myxococcus* spp. like ajudazol A, chloromyxamide, myxochromide A/C, rhizomide, anabaenopeptin NZ857/nostamide A, 1-heptadecene, alkylpyrone, myxalamide, bicornutin, dawenol, violacein, indigoidine, myxovirescin A1, phenalamide A2, fulvuthiacene A/B, and rhizopodin (Fig. S9B). Among predicted BGCs, ajudazol A in *M. faecalis* O35^T^, O15 (absent in *P. flavus* Y35^T^ and *P. hansupus* mixupus^T^) showed 53% and 61% similarity, respectively, to the ajudazol A biosynthetic gene cluster from *Chondromyces crocatus* (BGC0000954, AM946600.1) (Fig. S10A). A dawenol-synthesizing BGC in *M. faecalis* O35^T^ showed 88% sequence similarity (Fig. S10B) with *Stigmatella aurantiaca* DW4/3-1 (BGC0000044, CP002271.1), but no such cluster was present in *M. faecalis* O15 and *P. flavus* Y35^T^ and *P. hansupus* mixupus^T^. A dkxanthene coding BGC in *M. faecalis* O35^T^ and *P. flavus* Y35^T^ and *P. hansupus* mixupus^T^ showed similarity (>75%, Fig. S10C) with *Stigmatella aurantiaca* DW4/3-1 (BGC0000986, BN001209.1), whereas no such cluster was present in *Myxococcus faecalis* O15. Myxochelin synthesizing BGCs in *M. faecalis* O35^T^ and *P. flavus* Y35^T^ revealed 91% similarity (Fig. S10D) with *Stigmatella aurantiaca* Sg a15 (BGC0001345, AF299336.1) but was absent in *P. hansupus* mixupus^T^. Alkylpyrone-407 synthesizing BGCs in both *P. flavus* Y35^T^ and *P. hansupus* mixupus^T^ showed 56% similarity with alkylpyrone-407 biosynthetic gene cluster from a *Cystobacterineae* strain (BGC0001964, MH908886.1) (Fig. S10E). The myxoprincomide-c506 synthesizing BGCs from *P. flavus* Y35^T^-*P. hansupus* mixupus^T^ and *Myxococcus faecalis* O15-O35^T^ showed 100% and 66% similarity, respectively, with *Myxococcus xanthus* DK 1622 (BGC0000393, CP000113.1) (Fig. S10F).

*M. faecalis* O35^T^ and O15, though members of the same species, displayed differences in dkxanthene and dawenol-synthesizing gene clusters, indicating differences in genome composition reflecting intraspecies diversity. Furthermore, BGC alignment using the clinker tool revealed that the dkxanthene-synthesizing gene cluster had been rearranged in *M. faecalis* O35^T^ and *P. flavus* Y35^T^ (Fig. S10C). Thus, it might be possible that the rearrangement of biosynthetic genes occurred during speciation of *Myxococcus* spp. with respect to their ecological niche adaptation as observed in the fungal family (67). *P. flavus* Y35^T^ differed from *M. faecalis* O35^T^, O15, and other *Myxococcus* spp. in synthesizing alkylpyrone-407 and myxochromide A, whereas *M. faecalis* O35^T^, O15 possessed ajudazol and chloromyxamide, which were absent in *P. flavus* Y35^T^ and *P. hansupus* mixupus^T^. All these metabolites have been reported to possess antibacterial, antifungal, and antitumor activities (2, 68), which signifies that *Myxococcus* spp. are not only a rich source of bioactive compounds but may also play an important yet unknown role in gut homeostasis, as suggested by the isolation of fecal isolates described in this study.

#### Diversity and distribution of CAZymes

Apart from diverse bioactive compounds, *Myxococcus* spp. encoded a high number of CAZyme genes (167-234) involved in the degradation of complex carbohydrates into simpler sugars, which could be used as an energy source in microorganisms (69, 70). Genome analysis of *Myxococcus* spp. revealed genes coding for GH, GT, CBM, CE, AA, and PL that degrade complex carbohydrates (Fig. 9). GH and GT were the two most abundant CAZyme families that included GH13, GH23, GT2, and GT4 as the abundant domains (9). As in soil, these CAZymes might be involved in host physiological processes of digestion, nutrient absorption, and mucin catabolism (71), thus playing a crucial role in maintaining a healthy gut or preventing diseases (71–75). Starch is one of the major components of the human diet; however, a major fraction of the dietary fibre escapes host degradation and reaches the gut microbiota, and the CAZyme systems break down the polymer into monomeric and oligomeric units (76). CAZyme analysis of *M. faecalis* O35^T^ and O15, *P. flavus* Y35^T^ revealed the presence of starch, cellulose, and chitin-degrading domains (Fig. 9B; Table S4) that might play an important role in the digestion of polymers. Few GH13 domains were annotated as glycogen and trehalose synthesis (glycogen biosynthesis gene GlgB and trehalose biosynthesis gene) and breakdown (glycogen debranching gene GlgX) (Table S4). Several gut-colonizing bacteria have been reported to have genetic capabilities for glycogen metabolism, and a recent review suggested that glycogen metabolism helps gut bacteria to sustain in stress conditions and facilitates colonization (77–83). Apart from biomass degradation, myxobacterial CAZymes (GH16, GH18, GH19, and GH23) may also act in defense strategies and predation by being part of a secretory cocktail of antimicrobial compounds and lytic enzymes such as lysozyme and endolysins (33, 84). Zhao et al. (84) identified that GH18 and GH19 glycoside hydrolases from *Corallococcus* sp. EGB harboring chitinase activity plays a pivotal role as biocontrol agent (85). Li et al. (85) and Arend et al. (86) identified endolysins and lysozyme activities in GH19 from *C. silvisoli* C25j21 and *M. xanthus* DK1622, respectively (85, 86), important for its predatory activities against phytopathogenic fungi and bacteria, though only chitinase activity could be identified for the enzymes in the novel species (Table S4). Further CAZyme domains pertaining to lytic enzymes (GH18, GH19) related to predation might be very crucial for maintaining a healthy gut through the structuring of microbial populations, as has been recently shown from soil as well as from plant ecosystems (87, 88). Among glycosyl transferases, GT2 and GT4 are abundantly present (20–33) across the *Myxococcus* spp. and the Kyoto Encyclopedia of Genes and Genomes analysis suggested involvement in metabolic pathways (map01100) and cell wall biosynthesis like exopolysaccharide biosynthesis (map00543), N-glycan biosynthesis (map00510), and lipopolysaccharide biosynthesis (map00540) ( Table S7).

### Microbial ecology of gut isolates

Gut dysbiosis is closely associated with the pathogenesis of IBD, and it is of major interest to understand the etiology of the disease. The gut microbiome of IBD patients has reduced *Bacteroidetes* and *Firmicutes,* abundant *Proteobacteria* (89), and low amounts of beneficial bacteria *Ruminococcus, Faecalibacterium, Dorea, Blautia, Christensenellaceae, Collinsella, Roseburia,* and other obligate anaerobes involved in producing short-chain fatty acids (90, 91). However, the exact factors driving the alteration of resident microbial dynamics in IBD are still unclear (92–94). It has also been observed that detrimental and proinflammatory bacterial groups like *Enterobacteriaceae, Fusobacterium, Campylobacter*, *Megasphaera, Enterococcus,* sulfate-reducing *Gammaproteobacteria,* and *Deltaproteobacteria* were abundant in the IBD patients' stool and mucosa samples (95, 96). Bacteria in a natural environment are subjected to predation by *Bdellovibrios* and myxobacteria, bacteriophages, and unicellular *Eukaryotes* like protists; this is in fact one of the mechanisms proposed to maintain gut homeostasis (87, 97). Apart from *Bdellovibrio*, myxobacteria are one of the major predatory bacteria isolated broadly from soil and also from faeces of several herbivorous animals; however, their association with human hosts is poorly explored. Very few myxobacterial OTUs from human skin and one OTU from IBD patients have been reported (6, 14). A recent RNA-sequencing-based taxonomic profiling of CD and non-IBD control samples identified *Corallococcus exigus* in colonic biopsy samples of healthy controls, though few disease samples also displayed flares in the population of this taxa (98). Keeping this perspective in background, combined with the fact that myxobacteria are ubiquitous in their bio-geographic distribution (6, 99), we hypothesized that they could be an important yet under-reported group of the gut microbial community and focused on their isolation and detection through meta-barcoding sequencing from human fecal IBD samples. A wide diversity of myxobacterial OTUs were detected, and they were also present in one of the control samples (IMS1) (Fig. S6). A similar finding has been reported by Jacobsen et al. (98), who detected myxobacterial taxa in biopsy samples. Their study suggested that the relative abundance of family *Myxococcaceae* was higher in healthy control samples. This indicates that myxobacteria may be normal residents of the human gut microbiota but are grossly underrepresented, whose presence is flared during inflammatory diseases like other δ-*Proteobacteria* such as *Bdellovibrios* spp. (21, 100). Though it was difficult to rationalize the presence of the genus *Pseudenhygromyxa*, a typically marine myxobacterium in the human gut; however, Kaakoush et al. (14) detected similar OTUs related to the genus *Haliangium* in CD patients (14). Shatzkes et al. (22) observed the detection of OTUs belonging to *δ-Proteobacteria* and *Myxococcales* in animal models inoculated through the intrarectal route with predatory *Bdellovibrio bacteriovorus* (22). Thus, together with maintaining the soil nutrient cycle, these *Myxococcus* spp. might play an important hitherto unknown ecological equalizer role in the human gut through preying using their elaborate set of CAZymes, as has been shown recently in several myxobacteria-plant pathogen/soil interactions (80, 88). The presence of trehalose synthesis CAZymes in the novel strains was interesting, as few recent reports pertaining to both *in vivo* and *in vitro* models have associated the molecule with reduction of inflammatory markers and modulation of microbiota to prevent the incidence of *C. difficile* infection (101, 102); however, due to lack of sufficient evidence (more sequences/isolates), it is not feasible to speculate on their role in IBD pathogenesis and gut homeostasis. Since bacterial secretion systems play a crucial role in pathogenesis and virulence, these systems were further analyzed in the novel strains. Genomic analysis revealed that all three novel strains encoded intact T1SS and T2SS secretion systems, while Sec-SRP, Tat, T3SS, and T6SS* were present in a degenerated form, and T4SS and T5SS were completely absent (Fig. S11). Studies show that T2SS, T3SS*, Sec-SRP, and the Tat pathway are involved in bacterial predation, while T6SS was associated with kin selection in *Myxococcus xanthus* (103–107). Additionally, T6SS has been identified as a virulence mechanism that can disrupt host microbial communities by outcompeting beneficial microbiota, leading to reduced microbial diversity—a characteristic often associated with IBD pathology (108). However, these newly isolated strains from IBD fecal samples encoded a degenerated T6SS, supporting our hypothesis that these might be normal gut residents rather than being associated with pathogenesis. Together with T2SS, T3SS*, Sec-SRP, and Tat apparatus, they prey on gut microbiota and probably facilitate in maintaining gut homeostasis. Moreover, despite our successful isolation of the taxa as well as retrieval of similar OTUs from 16S rRNA gene tag sequencing from human fecal samples in this study as well as from previous studies (14, 98), myxobacterial genomes from human-related samples were absent in SRA metagenome data sets. This discrepancy highlights the importance of culture-dependent study, especially for capturing rare or difficult-to-culture taxa. It would be prudent to deeply explore and characterize these systems through other multi-omics-based approaches, such as whole genome metagenome sequencing/meta-transcriptomics, to fully appreciate their importance in this important niche.

### Description of *Pseudomyxococcus* gen. nov.

Pseu.do.my.xo.coc'cus. Gr. masc. adj. pseudes, false; N.L. masc. n. *Myxococcus*, a bacterial genus name; N.L. masc. n. *Pseudomyxococcus*, false *Myxococcus*.

The description of the genus is the same as that for its sole genus *Myxococcus* given by Lang and Stackebrandt (109) until now. No differentiating phenotypic characteristics from genus *Myxococcus* have been observed till now. The genus represents a distinct monophyletic lineage as supported by core genes-based tree along with overall genome relatedness parameters of PoCP and AAI.

The genome size ranges from 8.9 to 9.5 Mb, and genomic G+C content is 68.8–70.6 mol%.

The type species is *Pseudomyxococcus virescens* NBRC 100334^T^ (=DSM 2260^T^) (110).

The genus currently comprises of the type species *Pseudomyxococcus virescens* DSM 2260^T^ along with five other species: *Pseudomyxococcus hansupus* mixupus^T^, *Pseudomyxococcus xanthus* DSM 16526^T^, *Pseudomyxococcus vastator* AM301^T^, *Pseudomyxococcus macrosporus* Ccm8^T^, and *Pseudomyxococcus flavus* Y35^T^.

### Description of *Pseudomyxococcus flavus* sp. nov.

fla'vus. L. masc. adj. *flavus*, yellow.

Vegetative cells are Gram stain negative bacilli, measuring 0.42–0.46 × 2.39–3.14 µm, and myxospores are round in shape, measuring 1.27–1.39 µm in diameter, and display gliding motility on the agar surface. The colonies exhibited transparent swarming growth and formed a halo on the VY/2 agar around the colonies. Fruiting bodies appear yellow in color on VY/2 agar and form soft, slimy mounds, knob-like structures without stalks. Aerobic growth was observed at 25°C–40°C (optimum at 28°C) and pH at 6.0–9.0 (optimum 8.0–9.0) but not at pH 5.0 and 2.0% NaCl (optimum 1%). Positive for gelatin, tween 20 and 80, casein hydrolysis but not for esculin (in plate assay), starch and xylan. The major fatty acids are anteiso-C_15:0_, iso-C_15:0_, and anteiso-C_17:0_. Cells prey upon *Staphylococcus aureus*, *Escherichia coli*, *Klebsiella pneumoniae*, *Shigella boydii, Pseudomonas aeruginosa*, and *Vibrio cholerae*.

The genomic DNA G+C content is 69.9 mol% as calculated from the whole genome sequence. (GenBank accession number CP143624). The dDDH and ANI values are, respectively, 34.7% and 88.1% to its phylogenetically closest neighbor *P. hansupus* mixupus^T^. The type strain *P. flavus* Y35^T^ (=MTCC 15055^T^) was isolated from an IBD patient’s fecal sample.

### Description of *Pseudomyxococcus hansupus mixupus* sp. nov. comb. nov.

N.L. gen. masc. n. *hansupus*, named after Dr. Hans Reichenbach.

Vegetative cells are Gram stain negative bacilli, measuring 0.40–0.49 × 2.74–4.38 µm, and myxospores are round in shape, measuring 1.08–1.40 µm in diameter and move by gliding motility on agar surface. The colonies exhibited transparent swarming growth and formed a halo on the VY/2 agar around the colonies. Fruiting bodies appear pink in color on VY/2 agar and form soft, slimy mounds, knob-like structures without stalks. Aerobic growth was observed at 25°C–40°C (optimum 28°C–30°C) and pH at 6.0–9.0 (optimum 8.0–9.0) but not at pH 5.0 and 2.0% NaCl (optimum 1%). Gelatin, tweens 20 and 80, and casein are hydrolyzed, but not esculin (in plate assay), starch and xylan. The major fatty acids are anteiso-C_15:0_, anteiso-C_14:0_, anteiso-C_17:0_, and iso-C_11:0_ 3OH. Cells prey upon *Escherichia coli*, *Staphylococcus aureus*, *Pseudomonas aeruginosa*, *Shigella boydii,* and *Candida albicans*.

The genomic DNA G+C content is 69.1 mol% as calculated from the whole genome sequence. (GenBank accession number CP012109.1). The dDDH and ANI values are, respectively, 62.1% and 80.2% to its phylogenetically closest neighbor *M. fulvus* DSM 16525^T^. The type strain *Pseudomyxococcus hansupus* mixupus^T^ (=MTCC13553^T^) was isolated as a contaminant from a plate of *Chondromyces apiculatus* DSM 436^T^ during subculturing (27).

### Description of *Pseudomyxococcus virescens* comb. nov.

vi.res’cens. L. part. adj. virescens becoming green.

The description is similar to that given originally by Thaxter (110) and emended by Lang et al. (111). The accession numbers of the 16S rRNA gene and whole genome are DQ768119 and FNAJ01000056.1, respectively. The type strain is *P. virescens* M22^T^ (=DSM 2260^T^ =ATCC 25203^T^).

### Description of *Pseudomyxococcus vastator* comb. nov.

vas.ta’tor L. masc. n. vastator the ravager, after its ability to devastate colonies of prey cells.

The description is similar to that given by Chamber et al. (23). The accession numbers of the 16S rRNA gene and whole genome are JAAIYB01 and JAAIYB010000769, respectively. The type strain is AM301^T^ (=NCCB 100768^T^ =NBRC 114352^T^).

### Description of *Pseudomyxococcus xanthus* comb. nov.

Xan’thus. Gr. adj. xanthos yellow; M.L. masc. adj. xanthus yellow.

The description is similar to that given by Beebe (112). The accession numbers of the 16S rRNA gene and whole genome are AB218205 and FNOH01000000, respectively. The type strain is FB^T^ (=NCIB 9412^T^ =DSM 6796^T^)

### Description of *Pseudomyxococcus macrosporus* comb. nov.

ma.cro.spor’us. Gr. adj. makros long, large; Gr. masc. n. sporos seed; M.L. masc. adj. macrosporus with large spores.

The description is similar to that given originally by Reichenbach (28) emended by Chamber et al. (23). The accession numbers of the 16S rRNA gene and whole genome are AJ233921 and CP022203, respectively. The type strain is Cc m8^T^ (=DSM 14,697^T^ =CIP 109128^T^).

### Description of *Myxococcus faecalis* sp. nov.

fae.ca'lis. L. fem. n. faex (gen. faecis), dregs; L. masc./fem. adj. suff. -alis, suffix denoting pertaining to; N.L. masc. adj. faecalis, pertaining or relating to faeces.

Vegetative cells are Gram stain negative bacilli, measuring 0.41–0.69 × 2.91–4.56 µm and myxospores are round in shape, measuring 1.22–1.45 µm in diameter, and are motile by gliding on agar surface. Colonies exhibit transparent swarming growth and form a halo on VY/2 agar. Fruiting bodies appear pink in color on VY/2 agar and form soft, slimy mounds, knob-like structures without stalks. Aerobic growth was observed at 25°C–40°C (optimum 28°C–37°C) and pH at 6.0–9.0 (optimum 9.0) but not at pH 5.0 and 2.0% NaCl. Positive for hydrolysis of esculin, gelatin, tweens 20 and 80, and casein, and negative for starch and xylan. The major fatty acids are iso-C_15:0_, anteiso-C_15:0_, and iso-C_17:0_. Cells prey upon *Staphylococcus aureus*, *Shigella boydii, Escherichia coli*, *Klebsiella pneumoniae*, *Pseudomonas aeruginosa*, and *Vibrio cholerae*.

The genomic DNA G+C content is 70.1 mol% as calculated from whole genome sequencing (GenBank accession number CP144336). The dDDH and ANI values, respectively, are 62.5% and 95.5% to its phylogenetically closest neighbor *M. fulvus* DSM 16525^T^. The type strain *M. faecalis* O35^T^ (=MTCC 15054^T^) and *M. faecalis* O15 were isolated from an IBD patient’s fecal sample.

## MATERIALS AND METHODS

### Sample details

Fecal samples were collected from two patients designated as IBD35 and IBD15, diagnosed with ulcerative colitis at the Post Graduate Institute of Medical Education and Research (PGIMER), Chandigarh. IBD35 was a 47-year-old female, and IBD15 was a 41-year-old female.

### Isolation of myxobacteria

Isolation and purification were carried out by the *E. coli* baiting technique (113). Briefly, overnight grown *E. coli* DH5α were cross-streaked on water (WAT) agar supplemented with 50 mg/L cycloheximide. The cycloheximide (12.5 mg/L) treated pea-sized fecal sample was placed in the center of the *E. coli* growth streak and incubated for 1–3 weeks at 30°C. During the course of incubation, pink colonies isolated from samples IBD35 and IBD15 were designated as strains O35 and O15, respectively, and yellow-colored colonies isolated from IBD35 as strain Y35 that were purified through subsequent sub-culturing on VY/2 agar (g/L: Baker’s yeast, 5; CaCl_2_.2H_2_O, 1; Agar, 15; supplemented with cycloheximide, 50 mg/L; vitamin B12, 0.5 mg/L) every 1–2 weeks till a pure colony was visualized and confirmed through microscopy. Stereo microscopic observations were done at 2–3 days intervals for confirmation of fruiting body formation. The strains were preserved separately in 20% (vol/vol) glycerol and in CAS medium (Casitone—1%, MgSO_4_.7H_2_O—0.1%) (28) at −80°C in 2 mL vials (Tarsons: Cat no. 523053).

### Phenotypic characterization

The phenotypic tests were determined after cultivation on VY/2 for strains O35, Y35, O15, and *M. fulvus* DSM 16525^T^ and SP media (raffinose 1 g/L, sucrose 1 g/L, galactose 1 g/L, soluble starch 5.0 g/L, casitone 2.5 g/L, MgSO_4_ × 7H_2_O 0.5 g/L, K_2_HPO_4_ 0.25 g/L, Agar [Difco] 15.00) for *M. hansupus* mixupus for 5–7 days at 30°C. The morphological characteristics, i.e., fruiting bodies and swarms, were visualized under a stereo microscope (Nikon SMZ1500, 10×). Cells, myxospores, and Gram staining were performed as per established protocols (114) and examined through bright field microscopy (Olympus BX21, 1,000×). The scanning electron micrographs of vegetative cells were produced as per previously described (111). Cell suspensions from the exponential phase was collected on a 0.2 µm isopore membrane filter (Sigma-Aldrich) and fixed with fixative solution (2.5% glutaraldehyde and 2.5% paraformaldehyde) at 4°C overnight. Then, it was dehydrated through a graded ethanol series (30%, 50%, 70%, 80%, 90%, and 100%) for 20 min, followed by dehydration in a silica desiccator overnight. Samples were then coated with gold nanoparticles and observed in a scanning electron microscope (JEOL JSM-6010 PLUS-LS, JEOL Ltd., Japan) (115). Transmission electron microscopy (TEM) was performed for the morphology of myxospores. Briefly, one drop of phosphate-buffered saline was deposited on a carbonated copper grid and allowed to dry for 10 min, followed by uranyl acetate treatment. Finally, samples were air-dried and observed under an electron microscope (JOEL JEM-2100 high-resolution analytical TEM). Predation was quantified through lawn-based assay by measuring the zone of diameter as per Morgan et al. (30) and Livingstone et al. (116) using Gram-positive and Gram-negative taxa as prey cells (33, 116). Oxidase and catalase tests were, respectively, performed using discs impregnated with N-N-dimethyl-p-phenylenediamine (Hi-Media) and 3% (vol/vol) H_2_O_2_ solution. Physiological growth parameters at different temperatures, pH, and salinities (NaCl) were observed by visual scoring on VY/2 and SP agar plates. Briefly, a small patching was done in a round shape, and growth expansion was recorded on the basis of diameter, depth, and color after 7 days. Higher growth expansion is denoted to fast growth, followed by moderate and slow growth (8, 23). Hydrolysis of various polymers was performed according to recommended procedures (114, 117) using CY basal media (casitone 3 g/L, yeast extract 1 g/L, CaCl_2_.2H_2_O 1 g/L). For assessing antibiotic susceptibility, zone diameters were recorded on VY/2 or SP (pH 7.8) plates with antibiotic disc kits (Hi-Media Cat no. IC007-1PK) after incubation at 30°C for 7 days. Acid production from carbohydrates was checked on CY media with phenol red indicator supplemented with filter-sterilized substrate (2.5 mg/mL) in 5 mL test tubes (in triplicates). The API 20NE and API ZYM kit tests (BioMerièux) were conducted as per manufacturer’s instructions except that MD1 medium (CaCl_2_.2H_2_O 0.7 g/L, Casitone 3 g/L, MgSO_4_.7H_2_O 2 g/L, vitamin B12 0.5 g/L) was used for preparation of suspension inoculum fluid as per Stackebrandt et al. for API 20NE tests (43). For determination of cellular fatty acids, cells were grown on CY media plates to late log phase at 30°C for 5 days. The late log phase grown cells were then subjected to saponification, methylation, extraction, and washing according to the instructions of the MIDI system (MIS operating manual version 6.1; Sasser [118]). The samples were injected into an Agilent model 7890 gas chromatograph equipped with an autosampler and a flame ionization detector fitted with a 5% phenylmethyl silicone column (0.2 × 25 m). The injection-port temperature was 300°C, and the oven temperature was set between 170°C and 300°C, increasing at a rate of 1°C min^−1^.

### Genome sequencing and phylogenetic analyses

The genomic DNA was extracted according to the standard protocol using Zymogen Research Bacterial/Fungal DNA isolation kit (Zymo research) for small scale genomic DNA and Gentra Puregene kit (Qiagen) for large scale genomic DNA. The quality and quantity were confirmed by agarose (0.7% wt/vol) gel electrophoresis and Nanodrop spectrophotometer (Nanodrop 1000, Thermo Scientific). The protocols and conditions for 16S rRNA gene amplification, purification, and sequencing were followed as per Ojha et al. (119) except that purification of PCR products was done using a Qiaquick PCR purification kit (Qiagen) and sequencing completed using the primers 1100R (5'GGGTTGCGCTCGG-TTG3'), 518R (5'ATTACCGCGGCTGCTGG3'), and 926F (5'AAACTCAAAGGAATTGACGG3') on a ABI 3730xl genetic analyzer using the Applied Biosystems chemical guide second edition manual of DNA sequencing by capillary electrophoresis (https://tools.thermofisher.com/content/sfs/manuals/cms_041003.pdf). Whole genome sequencing was determined using a combination of long reads, i.e., Oxford Nanopore (Oxford Nanopore Technologies, Oxford, UK)/PacBio (Pacific Bioscience, USA) and short-read Illumina platforms (Illumina Inc., USA). Genomic DNA library preparation for PacBio SMRT sequencing technology was carried out as per the standard instructions in the protocol “Preparing Multiplexed Microbial Libraries Using SMRTbell Express Template Prep Kit 2.0” (https://www.pacb.com/wp-content/uploads/Procedure-Checklist—Preparing-Multiplexed-Microbial-Libraries-UsingM-RTbell-Express-Template-Prep-Kit-2.0.pdf, Part Number 101-696-100 Version 07, July 2020). The Oxford nanopore sequencing libraries were constructed using native barcoding expansion 1-12 (EXP-NBD104) and 13-24 (EXP-NBD114) and Ligation sequencing kit (SQK-LSK109) (Version: NBE_9065_v109_revAH_14Aug2019) and sequenced on an ONT PromethION sequencer. For Illumina sequencing, library preparation was performed with NEBNext Ultra DNA library prep kit (NEB #E7370S/L, Version 5.0) and sequenced on Illumina Novaseq 6000 platform. Using fastp with the default parameters, the reads were preprocessed and filtered (https://github.com/OpenGene/fastp). Filtlong was used to filter the nanopore reads (https://github.com/rrwick/Filtlong) with “keep percentage 80” and “target base 3 Gb” and the hybrid assembly of strain O35 was carried out using Trycycler (v0.5.0) (120), and for strains O15 and Y35, Flye (v2.9) (https://github.com/fenderglass/Flye) was used. Subsequent polishing of Flye-assembled genomes was performed using Medaka (v1.6.0) (https://github.com/nanoporetech/medaka), Polypolish (v0.5.0) (https://github.com/rrwick-/Polypolish), and Polca (v4.0.5) (121). Gene prediction and functional annotation were conducted using Bakta (v1.3.3) (https://github.com/oschwengers/bakta) with default parameters.

The 16S rRNA gene sequences of the strains O15, O35, and Y35 were retrieved from whole genome sequence and used as a query against the database of validly published prokaryotic names of type strains in EzTaxon server (http://www.ezcloud.net/eztaxon) to check closely related sequences and also to verify the authenticity and accuracy of the gene sequence obtained by PCR amplification. In order to construct phylogenetic trees, sequences from the closest reference taxa of the phylum *Myxococcata* were retrieved from GenBank and aligned with ClustalW2 (122). The evolutionary and phylogenetic analysis was performed using MEGA v7.0 (123). For other housekeeping genes, protein sequences were extracted from the genomes, and phylogenetic trees were generated using MEGA v7.0. The best substitution models were estimated by using the Maximum Likelihood statistical method implemented in the Model Selection Tool (Find Best DNA/Protein Models) within MEGA. For analysis of myxobacterial OTUs, the raw 16S rRNA gene-based meta-barcoding sequence files of fecal (UC remission-2, UC active-6 and control [non-IBD]-2) and biopsy (UC remission-8, UC active-20, and control [non-IBD]-13) samples were processed for OTU generation and identification with the standalone RDP classifier version 2.13 (≥97% sequence similarity) (124). The extraction and analysis of myxobacteria-specific OTUs were performed in the MEGAN community edition tool (version 6.24.10) (125). The phylogenetic tree of strains O15, O35, Y35, and myxobacteria OTUs was constructed using MEGA v7.0 (123). Additionally, the genomes of three strains, O15, O35, and Y35, were searched against metagenomic data sets in SRA using the mastiff implementation (https://github.com/sourmash-bio/mastiff) of sourmash brachwater (126, 127). The downstream analysis was conducted using Jupyter notebook (https://github.com/sourmash-bio/2022-search-sra-with-mastiff/blob/main/interpret-sra-live.ipynb).

To assess the phylogeny based on whole genome sequences, the FNA (nucleotide sequence) and FAA (protein sequence) files of the type strains of the *Myxococcaceae* family were retrieved from GenBank and used as input, respectively, in the UBCG pipeline (128) and PhyloPhlAn (129) to construct the trees based on 92 core genes and 400 marker proteins. The 35 universal single-copy marker genes from each selected genome (list of genes in Table S8) were aligned and concatenated using PhyloSuite (v1.2.3) (130). The ML tree was generated using the GTR (G+I) model with a bootstrap value of 100 using IQ-TREE (v1.6.8) integrated in PhyloSuite and visualized using iTOL (v5) (131). The best substitution models were estimated by using the Maximum Likelihood statistical method implemented in the Model Selection Tool (Find Best DNA/Protein Models) within MEGA. In order to calculate the overall genome identity, the parameters of GGDC that ascertains the dDDH or digital DNA-DNA hybridization, ANI, AAI, and POCP were measured, respectively, by Type (strain) Genome Server and GGDC 3.0 available at weblink (https://ggdc.dsmz.de/ggdc.php#) (33, 132), ANI calculator (133), average amino acid sequence identity tool (EzAAI) (134), and POCP calculator (https://github.com/hoelzer/pocp) among all the validly named species of *Myxococcaceae* family and strains O35, O15, and Y35. Functional categorization was performed using eggNOG-mapper (e value <1e-5) (v2) (135) by determining their COGs. Prediction of biosynthetic gene clusters (BGCs) was carried out using the antiSMASH tool (v6.0) (136) and further processed with BiG-SCAPE (https://github.com/medema-group/BiG-SCAPE). Among predicted BGCs, the synteny was further analyzed using Clinker (137). The antibiotic-resistant genes were identified using the ResFinder tool included in the Comprehensive Antibiotic Resistant Database (https://card.mcmaster.ca/analyze/rgi). CAZyme associated genes were identified in the dbCAN3 meta server, and potential hits were considered only when at least two of the three annotation tools integrated in dbCAN3 meta server (HMMER [e-value <1e-15, coverage >0.35], DIAMOND [e-value <1e-102], dbCAN_sub [e-value 1e-15, coverage >0.35]) identified them as potential CAzymes (138). Each of the CAZyme entries was manually verified by BLASTp (default parameters: identity ≥50%, coverage ≥50%, e-value cutoff <0.05) search against GenBank for confirming the function. All heatmaps were generated using TBtools-II (https://github.com/CJ-Chen/TBtools-II). Secretion systems were predicted using the MacSyFinder v2.0 (https://github.com/gem-pasteur/macsyfinder) program and BlastKOALA (https://www.kegg.jp/blastkoala).

## Data Availability

The complete genome sequences of strain O35, strain O15, and strain Y35 are available in GenBank under accession no. CP144336, CP144335, and CP143624, respectively. Figures S1 to S11 and Tables S1 to S8 are available at https://figshare.com/s/22757f23ba34b4ceb90a.
